# Bacterial biogeography of adult airways in atopic asthma

**DOI:** 10.1186/s40168-018-0487-3

**Published:** 2018-06-09

**Authors:** Juliana Durack, Yvonne J. Huang, Snehal Nariya, Laura S. Christian, K. Mark Ansel, Avraham Beigelman, Mario Castro, Anne-Marie Dyer, Elliot Israel, Monica Kraft, Richard J. Martin, David T. Mauger, Sharon R. Rosenberg, Tonya S. King, Steven R. White, Loren C. Denlinger, Fernando Holguin, Stephen C. Lazarus, Njira Lugogo, Stephen P. Peters, Lewis J. Smith, Michael E. Wechsler, Susan V. Lynch, Homer A. Boushey, for the National Heart, Lung and Blood Institute’s “AsthmaNet”

**Affiliations:** 1 Department of Medicine, Division of Gastroenterology, University of California San Francisco, San Francisco, CA USA; 2 Department of Internal Medicine, Division of Pulmonary/Critical Care Medicine, University of Michigan, Ann Arbor, MI USA; 3 Department of Medicine, Division of Pulmonary/Critical Care Medicine, University of California San Francisco, San Francisco, CA USA; 4 Department Microbiology/Immunology and Sandler Asthma Basic Research Center, University of California San Francisco, San Francisco, CA USA; 5 Division of Pediatric Allergy, Immunology and Pulmonary Medicine, Washington University School of Medicine, St Louis, MO USA; 6 Division of Pulmonary and Critical Care Medicine, Washington University School of Medicine, St Louis, MO USA; 7 Department of Public Health Sciences, Penn State University, Hershey, PA USA; 8 Department of Medicine, Brigham & Women’s Hospital, Boston, MA USA; 9 University of Arizona, Health Sciences, Tucson, AZ USA; 10 Department of Medicine, National Jewish Hospital, Denver, CO USA; 11 Department of Medicine, Northwestern University, Chicago, IL USA; 12 Department of Medicine, University of Chicago, Chicago, IL USA; 13 Department of Medicine, University of Wisconsin-Madison, Madison, WI USA; 14 The University of Pittsburgh Asthma Institute at UPMC/UPSOM, Pittsburgh, PA USA; 15 Duke Asthma, Allergy & Airway Center, Duke University School of Medicine, Durham, NC USA; 16 Wake Forest School of Medicine, Winston-Salem, NC USA

**Keywords:** Adult asthma, Atopy, Upper airways, Lower airways, Bronchial microbiota, Nasal microbiota, Induced sputum microbiota, Oral microbiota, Eosinophilic inflammation, *Moraxella*, *Corynebacterium*

## Abstract

**Background:**

Perturbations to the composition and function of bronchial bacterial communities appear to contribute to the pathophysiology of asthma. Unraveling the nature and mechanisms of these complex associations will require large longitudinal studies, for which bronchoscopy is poorly suited. Studies of samples obtained by sputum induction and nasopharyngeal brushing or lavage have also reported asthma-associated microbiota characteristics. It remains unknown, however, whether the microbiota detected in these less-invasive sample types reflect the composition of bronchial microbiota in asthma.

**Results:**

Bacterial microbiota in paired protected bronchial brushings (BB; *n* = 45), induced sputum (IS; *n* = 45), oral wash (OW; *n* = 45), and nasal brushings (NB; *n* = 27) from adults with mild atopic asthma (AA), atopy without asthma (ANA), and healthy controls (HC) were profiled using 16S rRNA gene sequencing. Though microbiota composition varied with sample type (*p* < 0.001), compositional similarity was greatest for BB-IS, particularly in AAs and ANAs. The abundance of genera detected in BB correlated with those detected in IS and OW (*r* median [IQR] 0.869 [0.748–0.942] and 0.822 [0.687–0.909] respectively), but not with those in NB (*r* = 0.004 [− 0.003–0.011]). The number of taxa shared between IS-BB and NB-BB was greater in AAs than in HCs (*p* < 0.05) and included taxa previously associated with asthma.

Of the genera abundant in NB, only *Moraxella* correlated positively with abundance in BB; specific members of this genus were shared between the two compartments only in AAs. Relative abundance of *Moraxella* in NB of AAs correlated negatively with that of *Corynebacterium* but positively with markers of eosinophilic inflammation in the blood and BAL fluid. The genus, *Corynebacterium*, trended to dominate all NB samples of HCs but only half of AAs (*p* = 0.07), in whom abundance of this genus was negatively associated with markers of eosinophilic inflammation.

**Conclusions:**

Induced sputum is superior to nasal brush or oral wash for assessing bronchial microbiota composition in asthmatic adults. Although compositionally similar to the bronchial microbiota, the microbiota in induced sputum are distinct, reflecting enrichment of oral bacteria. Specific bacterial genera are shared between the nasal and the bronchial mucosa which are associated with markers of systemic and bronchial inflammation.

**Electronic supplementary material:**

The online version of this article (10.1186/s40168-018-0487-3) contains supplementary material, which is available to authorized users.

## Background

A growing body of evidence implicates perturbations to the composition and function of bronchial bacterial communities in the pathophysiology of asthma across a broad breadth of clinical presentations [1–7]; relative enrichment of members of the *Proteobacteria*, such as *Haemophilus* and *Neisseria* in the lower airways, is a consistent feature of inflammatory airway disease [1, 3–5, 7]. We confirmed this enrichment in our recent study of mild atopic asthmatic subjects, in conjunction with bronchial expansion of the oral commensals, *Fusobacterium* and *Porphyromonas* [3], whose relative abundance was associated with sputum eosinophilia. Like most studies of the bronchial microbiota in asthma, our study relied on the invasive procedure of bronchoscopy. We used this procedure to obtain protected bronchial brushings, whereas others have used it to obtain unprotected brushings or broncho-alveolar lavage (BAL) for microbial analysis [1, 4]. Taken together, these independent observations have provided tantalizing insights into the relationships between the bronchial microbiota and asthma but suggest great complexity, with a highly heterogeneous microbial community differing across subjects with distinct phenotypes of asthma and possibly within subjects over time. Unraveling these complex relationships will likely require longitudinal studies of large numbers of subjects, but the costs, discomforts, and expertise required to perform bronchoscopy significantly limit subject recruitment. The studies done so far have thus mostly enrolled only adults and have been cross-sectional in design and small in size.

Correcting these deficiencies will require less invasive sampling approaches for studying the airway microbiome in asthma. Several studies have reported asthma-associated differences in bacterial community composition of sputum samples from asthmatic subjects [8–11], characterized by expansion of *Haemophilus*, *Moraxella*, and *Streptococcus*. Enrichments in these genera have also been described in studies of hypopharyngeal aspirates from children with wheezy respiratory illness [12], of hypopharyngeal and nasopharyngeal samples from infants who went on to develop asthma [13, 14], and in nasal samples of asthmatic children [15] including those who experienced more severe rhinovirus infections and related exacerbations of asthma [14, 16].

The associations of asthma and of asthma exacerbations with perturbations of the bacterial communities in the nose, nasopharynx, or induced sputum raise questions as to the relationships between the microbiota of the upper and lower airways. In healthy adults, dispersal of microorganisms from the oral (but not the nasal) cavity is considered the dominant source of bronchial microbiota [17–20], through naturally occurring microaspiration rather than carry over during sample collection [18]. It remains unclear, however, whether the bacterial topography of healthy airways is also characteristic of asthmatic airways. It is also unclear how representative the bacterial communities described in less-invasive sample types are of the composition of bronchial microbiota in asthma.

To address these questions, we took advantage of protected bronchial brush (BB), induced sputum (IS), oral wash (OW), and nasal brush (NB) samples obtained in a subset of a larger cohort of adults in whom the relationships between the bacterial microbiota in BB samples and disease category (atopy with mild asthma—AAs; atopy without asthma—ANAs; and neither atopy nor asthma—HCs) were studied [3]. We anticipated that the bacterial microbiota detected in the less-invasive sample types would differ from those found in BB samples, and we sought to address two main questions in our analysis: first, to determine which of these samples most closely resembled protected bronchial brushings in community composition, and, second, to determine which, if any, of these samples also revealed known asthma-related taxa or taxa associated with clinical or inflammatory features of atopic asthma in our subjects.

## Results

### Study group characteristics

Atopic asthmatic (AA) subjects in this cohort had mild well-controlled disease, significantly higher serum total IgE, blood and sputum eosinophil cell counts, and were more likely to report a history of allergic rhinitis compared to atopic non-asthmatics (ANAs) and non-atopic healthy control (HC) subjects (Table 1 and Additional file 1 Table S1).

**Table 1:** Study cohort characteristics

Variable	Allergic asthmatics (AA) (*n* = 22)	Allergic non-asthmatics (ANA) (*n* = 12)	Non-allergic non-asthmatics (HC) (*n* = 11)	*p* value^#^
Age (yrs)	39 (27–45)	33 (25–47)	28 (26–48)	NS
ACQ score (baseline)*	0.7 (0.3–1.0)	–	–	**–**
% Male	50%	67%	36%	NS^€^
% White	68%	50%	72%	NS^€^
BMI (kg/m^2^)	25 (23–30)	27 (21–32)	27 (23–28)	NS
FEV1% predicted pre-Alb^¥^	86 (69–97)	98 (91–107)	104 (97–112)	**0.001**
FEV1% predicted post-Alb^¥^	99 (83–106)	104 (96–107)	107 (101–121)	**0.035**
Change in FEV%	9.5 (6.0–15.0)	3.0 (1.0–6.5)	4.0 (1.0–5.0)	**< 0.0001**
PC_20_ (methacholine)	1.1 (0.3–2.8)	> 32^$^	> 32^$^	**< 0.0001**
Serum IgE (EU/mL)	169.5 (68.8–313.0)	88.5 (43.5–175.0)	14.0 (5.0–37.0)	**< 0.0001**
No. of positive sIgE^¢^	6 (3–9)	4 (2–6)	–	NS^&^
Allergic rhinitis (%)	55%	25%	0%	**0.006** ^€^
Blood neutrophils (%)	53.1 (48.0–61.2)	52.8 (51.0–61.8)	58.4 (53.3–63.0)	NS
Blood eosinophils (%)	3.7 (1.9–5.3)	2.0 (1.4–5.0)	1.8 (1.2–3.0)	*0.076*
Sputum neutrophils (%)	50.8 (30.1–63.6)	34.6 (15.0–44.4)	41.1 (37.7–65.5)	NS
Sputum eosinophils (%)	0.5 (0.0–2.6)	0.1 (0.0–0.5)	0.0 (0.0–0.4)	**0.041**
BAL GM-CSF (pg/mL)	349 (194–571)	476 (233–840)	154 (125–258)	**0.010**
BAL IL-6 (pg/mL)	102 (49–185)	160 (67–164)	70 (23–110)	NS
BAL IL-7 (pg/mL)	0.3 (0.3–37.6)	0.3 (0.3–20.5)	0.3 (0.3–0.3)	NS
BAL IL-8 (pg/mL)	1407 (843–3864)	1311 (870–2851)	953 (503–1180)	NS
BAL CXCL11 (pg/mL)	371 (137–628)	801 (421–937)	339 (171–396)	**0.036**
BAL MIP-1α (pg/mL)	82 (49–174)	114 (74–156)	69 (38–106)	NS
BAL MIP-1β (pg/mL)	317 (200–616)	275 (240–394)	128 (113–350)	*0.095*
BAL MIP-3α (pg/mL)	390 (162–1668)	411 (242–715)	264 (126–918)	NS
BAL TNF (pg/mL)	70 (17–99)	65 (42–106)	29 (22–76)	NS
BAL IL-1β (pg/mL)	0.04 (0.04–4.2)	0.04 (0.04–7.9)	0.04 (0.04–0.04)	NS
BAL IL-21 (pg/mL)	0.8 (0.04–17.8)	8.3 (0.04–37.1)	0.04 (0.04–3.1)	NS

All values are medians (IQR).*ACQ—Asthma Control Questionnaire. ^¥^Alb—Albuterol. ^$^Methacholine challenge was stopped at 32 mg/dL and PC_20_ for these subjects was censored. ^¢^Number of positive specific IgE (sIgE > 0.35 kU/l) from a total of 12 aeroallergens tested by ImmunoCap assay. Statistical significance was determined using ^#^Kruskal-Wallis, ^&^Mann-Whitney, or ^€^Chi-square test with *p* values > 0.1 assigned NS; *p* values <0.05 are highlighted in bold and those trending towards significance <0.1 are italicized

### The nasal airways harbor less complex bacterial communities than the lower airways

**Fig. 1:**
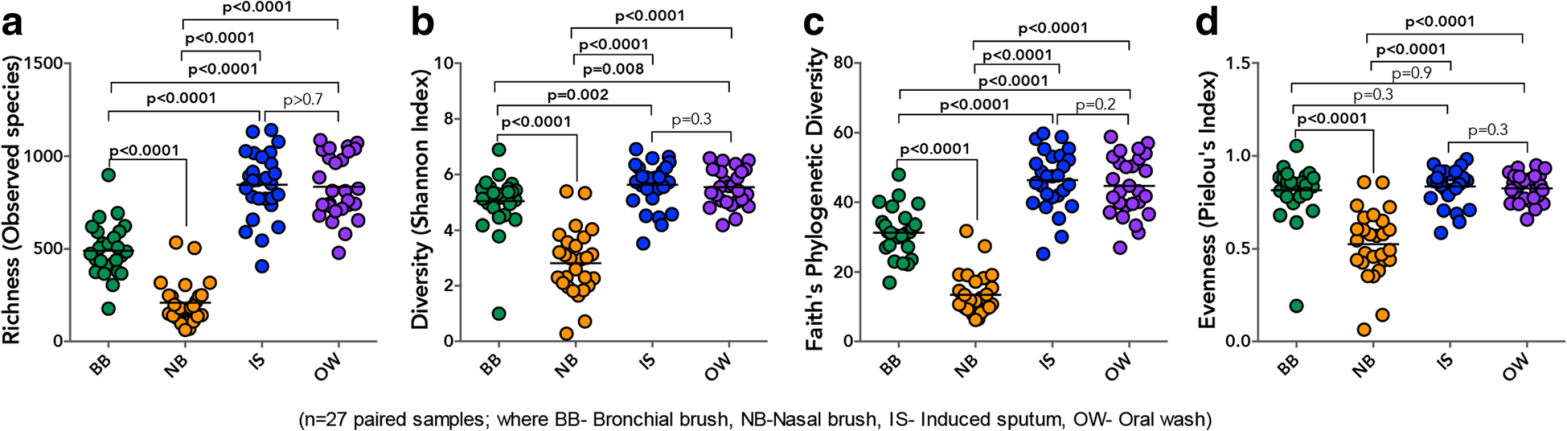
Alpha diversity in the microbiota of different specimen types demonstrating that the upper airway harbors significantly sparser bacterial communities than the lower airways or the oral cavity. **a** Bacterial richness as indicated by the total number of taxa detected in each sample type. **b** Shannon index of bacterial diversity in each sample type. **c** Phylogenetic Faith’s index of bacterial diversity in each sample type. **d** Pielou’s index of community evenness in each sample type. Statistical significance was determined using Wilcoxon matched-pairs signed rank test

To determine compositional similarity between less-invasive airway sample types and BBs, we first focused on alpha diversity indices for the subset of samples (*n* = 27 pairs) from our larger study cohort [3], for which NB samples were collected and all four samples were profiled (NB, IS, OW, and BB; Additional file 1: Table S2). Comparison of alpha diversity indices across these paired specimen types showed that the richness and diversity of the microbiota of BB was significantly higher than NB, but significantly lower than IS and OW (Fig. 1a–d). Expanding the comparison of alpha diversity indices between BB, IS, and OW to the larger subset of samples (i.e., including subjects from whom NB samples were not obtained; *n* = 45; Additional file 1: Table S2), confirmed that bacterial communities detected in IS samples were more diverse than both BB and OW (Additional file 1: Figure S1a–d). These observations suggest that the microbiota found in IS is likely sourced in variable degrees from the upper and lower airways. In contrast, the nasal airways comprise of less complex communities than those detected in the bronchi of adult subjects, findings that are in agreement with those from other studies [5, 17]. We additionally found the microbiota of IS to be more diverse than that of oral wash, indicating that this sample type reflects a more complex community. Importantly, this relationship in alpha diversity across sample types remained conserved in samples from AA and HC subjects analyzed independently (Additional file 1: Figure S2a–b).

As reported previously [3], phylogenetic diversity in the subset of BBs examined in this study (*n* = 27 and *n* = 45) tended to be higher in AAs compared to HC subjects (Additional file 1: Figure S2a–b). No such trend was observed in comparison of the other sample types from the same individuals, nor was a difference in other alpha-diversity indices found from comparison of AAs and HCs (data not shown). These findings suggest that the phylogenetic expansion reported in our earlier study of mild atopic asthma is confined to protected BB samples; the signature is not detected in other sample types from our very mild group of asthmatic adults.

### Bacterial microbiota in BB, IS, OW, and NB across all subjects is compositionally distinct

**Fig. 2:**
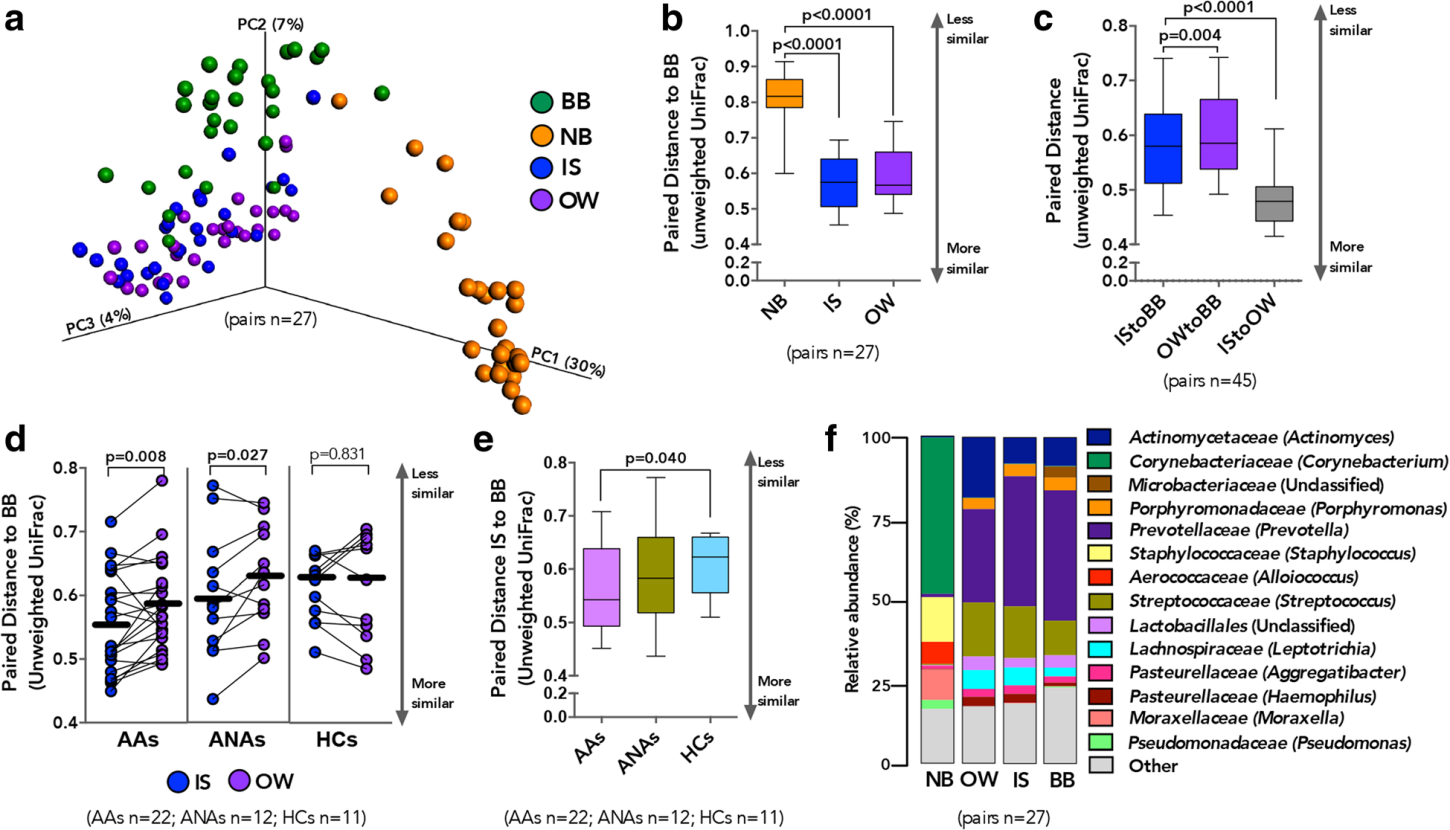
Compositionally lower airway microbiota is more similar to the oral than nasal cavity. **a** Principal coordinate analysis (unweighted UniFrac) shows compositional dissimilarity between paired samples (linear mixed-effects model, *p* < 0.0001). **b** Mean intra-subject paired distance to BB shows that the NB microbiota are most distinct from BB (Wilcoxon matched-pairs signed rank test; Whiskers extend to 95% confidence interval). **c** Shorter mean intra-subject paired distance between IS and BB compared to OW and BB, suggests that the IS microbiota are more representative of BB than OW is of BB (Wilcoxon matched-pairs signed rank test; Whiskers extend to 95% confidence interval). **d** Shorter mean intra-subject paired distance is observed between BB and IS compared to OW in AAs and ANAs but not HCs (Paired *t* test). **e** Shorter mean intra-subject paired distance between IS and BB in AAs compared to HCs (Welch’s corrected *t* test; Whiskers extend to 95% confidence interval) suggests that IS microbiota are more representative of BB in asthmatic subjects. **f** Summary of the relative abundance of taxa identified in paired samples (*n* = 27) shows NB as being the most compositionally distinct. Bacterial taxonomic classification is shown at a family (genus) level

We next assessed the overall phylogenetic community composition by principal coordinate analysis and found that microbiota composition co-varied with sample type (Fig. 2a; unweighted UniFrac; LME *p* < 0.001). Bronchial microbiota was compositionally distinct from all other sample types (Additional file 1: Figure S3a–c; unweighted UniFrac; LME *p* < 0.001), although intra-subject paired distance between BB and other sample types was significantly shorter for OW than NB (Fig. 2b and Additional file 1: Figure S4a), suggesting less compositional similarity between the microbiota of the bronchi and the nasal cavity. Bacterial microbiota membership (Fig. 2c) and to a lesser extent community structure (Additional file 1: Figure S4a–b) in BB were more similar to those detected in IS samples than between BB and NB or OW samples.

Using paired samples from 45 participants, we demonstrate that although the membership of the microbiota in IS resembled OW more closely than BB (unweighted UniFrac; Fig. 2c), this was not the case for the community structure (weighted UniFrac; Additional file 1: Figure S4b). Overall, bacterial communities detected in IS were distinct from OW samples (Additional file 1: Figure S3d; unweighted UniFrac LME *p* = 0.019), indicating that IS microbiota are not simply mirroring OW bacterial communities. To determine whether this observation was consistent across the three subject groups, we examined the mean paired distance between BB-IS and BB-OW across AAs, ANAs, and HCs. Mean paired distance to BB was shorter for paired IS than OW in AAs (unweighted UniFrac; Fig. 2d and weighted UniFrac; Additional file 1: Figure S4c), indicating that the resemblance in bacterial membership of IS to BB was greater in AA than in HC subjects (Fig. 2e). Community membership in IS samples of ANAs was also more similar to BB than those detected in OW (unweighted UniFrac; Fig. 2d) though this relationship did not extend to the overall community structure (weighted UniFrac; Additional file 1: Figure S4c). However, in the HC subjects, mean paired distance between BB-OW and BB-IS was indistinguishable (Fig. 2d and Additional file 1: Figure S4c). Unfortunately, likely due to the small number of HCs in whom NB samples were collected, confounded further by greater dissimilarity in community composition between NB and BB, we found no difference in the BB-NB paired distance between AAs and HC subjects (Additional file 1: Figure S4d–e).

### Taxonomic architecture in IS samples more closely resembles that of the bronchial microbiota in asthmatic than in healthy adults

Taxonomic comparison across samples (Fig. 2f) indicated that the overall relative abundance of bacterial genera detected in BB samples correlated more strongly with those detected in IS and OW (Pearson r median [IQR] = 0.869 [0.748–0.942] and *r* = 0.822 [0.687–0.909] respectively Additional file 1: Table S3) than with those present in the NB samples (*r* = 0.004 [− 0.003–0.011]; Additional file 1: Table S3 and Figure S5a). Consistent with our earlier observation of a shorter mean paired distance between BB-IS compared to BB-OW, taxonomic composition in BB correlated more strongly with those in IS than OW (Additional file 1: Figure S5b) in the AAs but not ANA or HC subjects (Additional file 1: Figure S5c–e). Together, these findings suggest that asthma and to a lesser extent atopy may be associated with heightened predisposition of the airways to bacterial colonization resulting in communities which are less like those colonizing the oral cavity.

**Fig. 3:**
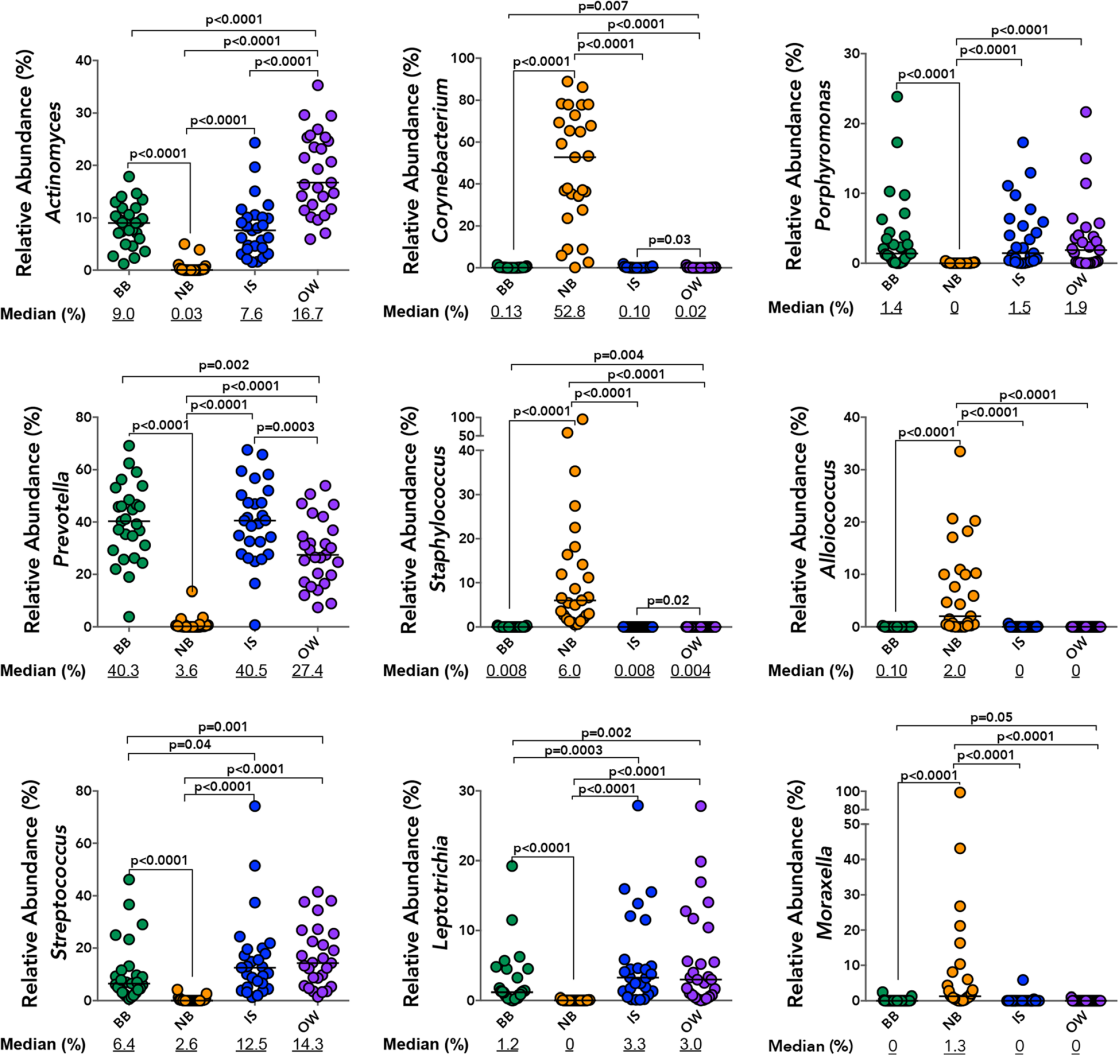
Distribution of the relative abundance for most prevalent genera (present at ≥ 3% in any one of the samples) in the four paired (*n* = 27) specimen types, illustrates the dissimilarity in bacterial composition of NB compared to other samples. Significance was determined using Wilcoxon matched-pairs signed rank test (*p* > 0.05 not shown)

The bronchial microbiota was predominantly colonized by *Prevotella, Actinomyces*, and to a lesser extent *Porphyromonas* and *Streptococcus* (Fig. 3), a pattern similar to that observed for IS samples. Specifically, the relative abundance of *Prevotella, Actinomyces, Corynebacterium*, and *Staphylococcus* was indistinguishable between paired BB and IS samples (Fig. 3) but differed between these two samples and the OW. Furthermore, the overall relative abundance of *Prevotella* and *Corynebacterium* correlated between BB and IS paired samples (Additional file 1: Table S4) as did those of *Porphyromonas*, *Streptococcus*, and *Leptotrichia*. Although the relative abundance of all highly prevalent genera examined (present at ≥ 3% in either of the samples) was distinct between BB and OW samples, a significant correlation was observed in the relative abundance of *Leptotrichia*, *Porphyromonas*, *Corynebacterium*, and *Streptococcus* (Additional file 1: Table S4). It should be noted, though, that the relative abundance of all highly prevalent genera examined (present at ≥ 3% in either of the samples) also correlated between IS and OW (Additional file 1: Table S4), attesting to the unsurprising similarity in community composition between these sample types. In contrast, NB samples were predominantly colonized by *Corynebacterium* followed by *Staphylococcus*, *Streptococcus*, *Alloiococcus*, and *Moraxella* (Fig. 3). Notably, the relative abundance of only *Moraxella* correlated strongly between BB and NB samples without also showing a correlation between BB-IS and BB-OW (Additional file 1: Table S4). No significant difference in the relative abundance of the above genera were observed in any of the four samples between AAs and HC subjects, although that of a *Staphylococcus* trended to be higher in NBs of AAs (Additional file 1: Figure S6a), This enrichment was confirmed at operational taxonomic unit (OTU) level in the NB of AA subjects (Additional file 1: Figure S6b).

**Fig. 4:**
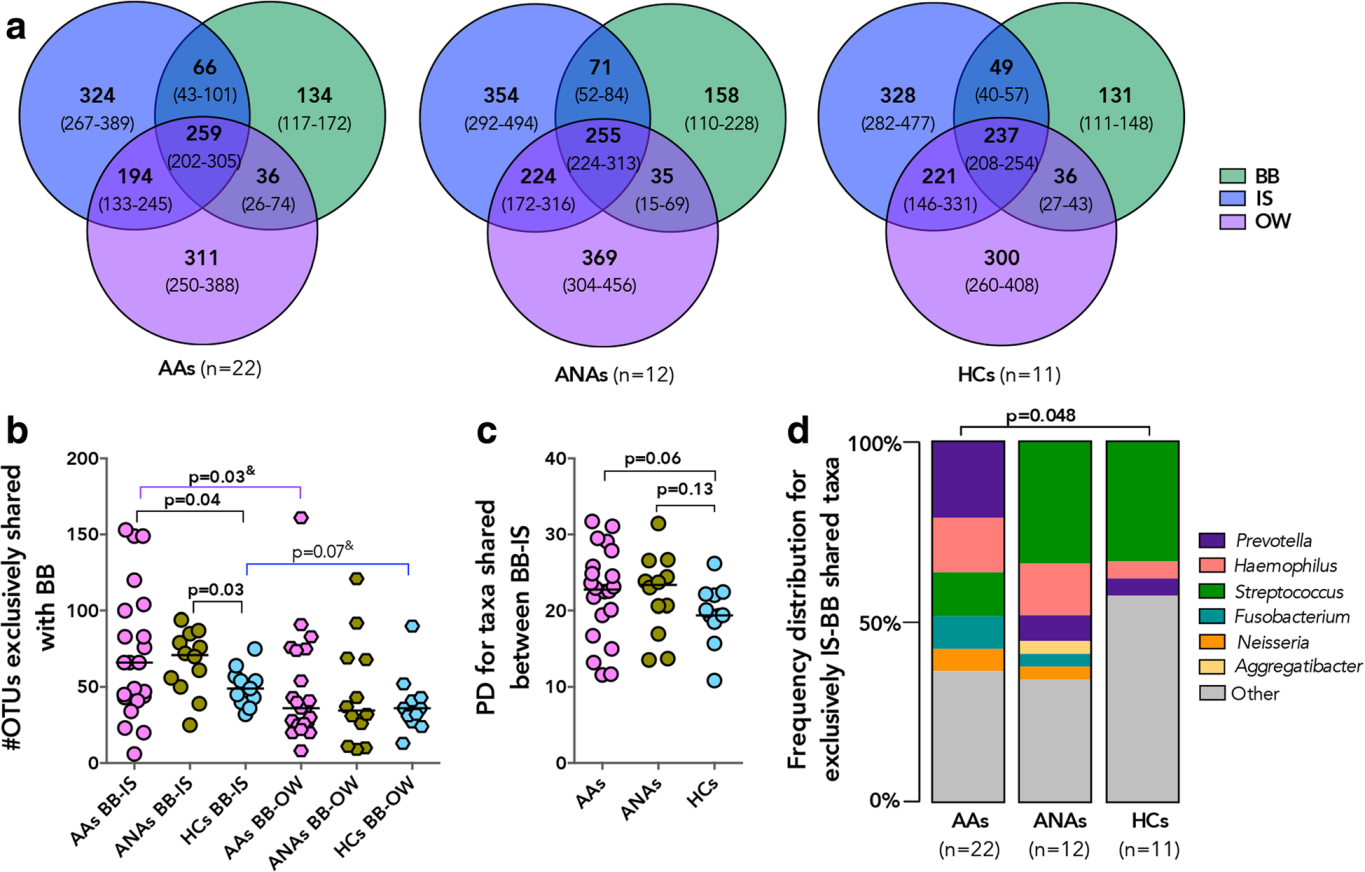
A greater number of operational taxonomic units (OTUs) was exclusively shared between IS-BB in AAs compared to HC subjects and included taxa previously associated with asthma. **a** Number of OTUs associated with specific sample type in AAs, ANAs, and HC subjects. Values are shown as a median (IQR). **b** A greater number of OTUs was shared exclusively (in at least 20% of subjects) between IS and BB in AAs and ANAs compared to HCs, which was greater than those shared in BB-OW (Welch’s corrected *t* test and ^&^Wilcoxon matched-pairs signed rank test; *p* > 0.2 not shown). **c** Faith’s Phylogenetic diversity (PD) of taxa shared between paired IS-BB samples trended to be higher in AAs and ANAs compared to HCs (Mann Whitney test; *p* > 0.2 not shown). **d** Frequency distribution of specific genera (present in at least 20% of participants for each group) for OTUs shared exclusively between IS-BB was distinct in AAs and HCs and included known asthma-associated taxa (Chi-square test; *p* > 0.05 not shown)

Next, we evaluated the number of specific OTUs per participant in a larger number of samples (BB, IS, and OW *n* = 45 pairs), which were exclusive or shared across sample types in our three subject groups (Fig. 4a). We found that a large and similar number of OTUs was shared between all three sample types in all subject groups (Kruskal-Wallis *p* = 0.48), but that the median number of OTUs shared between IS-BB but not between OW-BB samples was higher in AAs and ANAs compared to HC subjects (Fig. 4b). Interestingly, within the AAs, the number of IS-BB-shared OTUs was higher than those shared between OW-BB paired samples (Fig. 4b). Additionally, phylogenetic diversity for taxa shared between IS-BB samples trended to be higher in AAs and ANAs compared to HCs (Fig. 4c), which was not observed for taxa shared between OW-BB samples (Mann Whitney; *p* > 0.2). The OTUs predominantly shared (in at least 20% of subjects) between BB-IS samples in AAs included members of *Prevotella* and a number of other genera previously reported to be associated with asthma, specifically *Haemophilus*, *Fusobacterium*, and *Neisseria* [3], which were not shared at the same frequency between BB-IS samples in the HC subjects (Fig. 4d). Overall, these findings suggest that though significant differences are apparent between all non-invasive sample types and protected bronchial brush bacterial communities, IS microbiota more closely resemble that of BB than do those in OW and NB samples in atopic asthmatic and atopic non-asthmatic adults. This is not to say that taxa detected in IS represented all those detected in the BB, since a number of taxa detected in each sample included sample-specific members of *Fusobacterium*, *Neisseria*, *Aggregatibacter*, *Streptococcus*, and *Prevotella* (Additional file 1: Figure S5f). Additionally, among taxa identified in BB but not IS were members of genera whose enrichment has previously been associated with asthma [1, 3, 7, 9], such as *Pseudomonas*, *Sphingomonas*, and *Moraxella*; this was true as well for *Lactobacillus* whose depletion has been similarly associated with asthma [3]. Conversely, among taxa detected in IS that were not detected in BB were specific members of *Haemophilus* and *Porphyromonas* (Additional file 1: Figure S5f), whose enrichment has been reported in asthmatic airways in previous studies.

### Bronchial microbiota comprise taxa shared with both the oral and to a lesser extent the nasal cavity and include a suite of asthma-associated bacterial genera

**Fig. 5:**
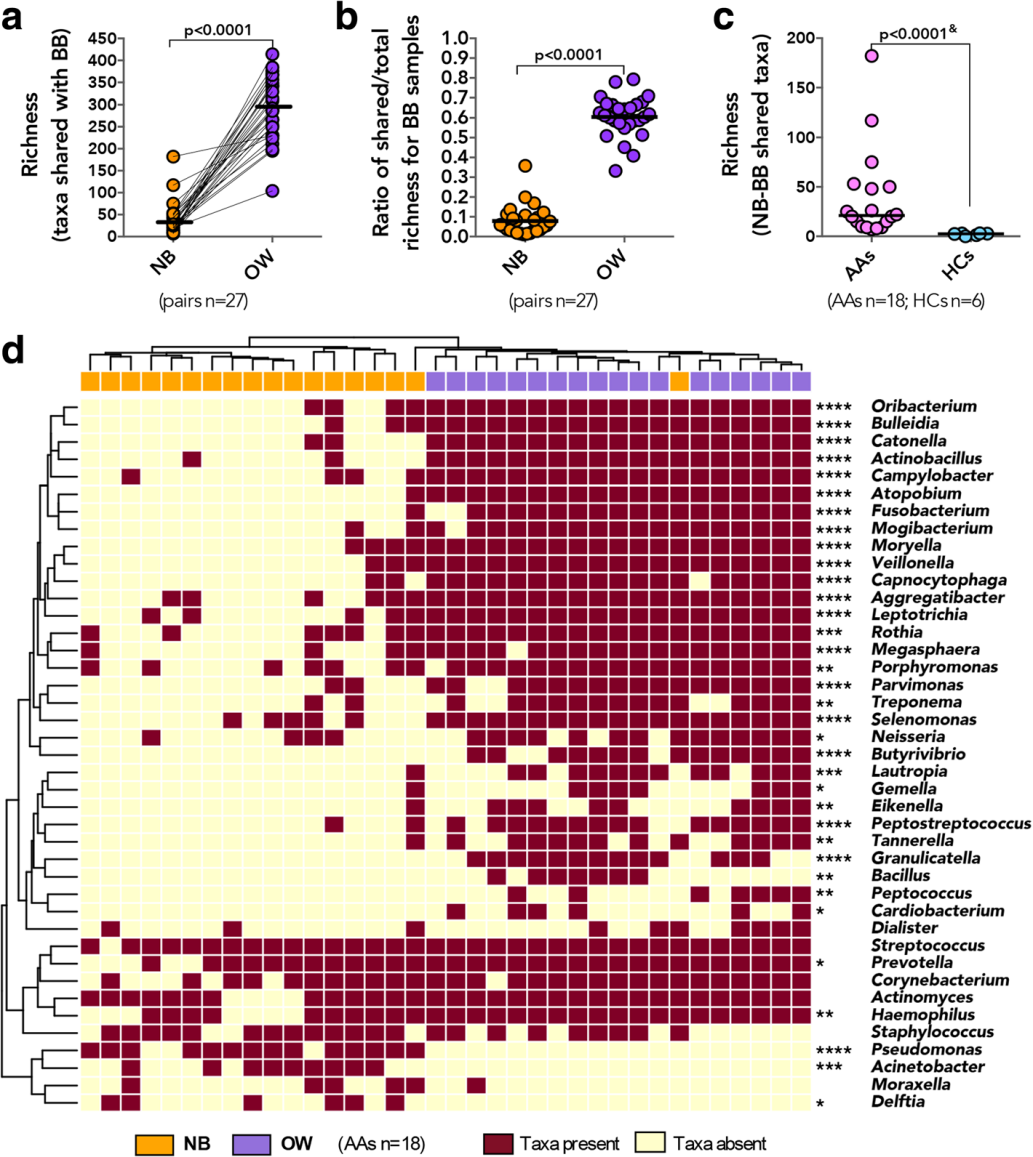
A proportion of all the taxa detected in the BB is shared with paired NB and OW samples, with each compartment contributing distinct taxa to the bronchial community. **a** The number of taxa shared with BB is higher for OW compared to NB (Wilcoxon matched-pairs signed rank test). **b** Richness of taxa shared represents a larger proportion of the overall richness detected in BB sample for paired OW compared to NB (Wilcoxon matched-pairs signed rank). **c** Richness of the shared taxa between paired BB and NB was greater in AAs compared to HCs (^&^Mann-Whitney test). **d** Distribution of specific taxa shared with paired BB is distinct between NB and OW samples in AAs. (Genera identified in at least 20% of subjects in the same sample type; Statistical significance between OW and NB genera was determined using Fisher’s exact test where ****p ≤ 0.0001, ***p ≤ 0.001, **p ≤ 0.01, *p ≤ 0.05)

Considering that the bronchial microbiota in healthy subjects has been reported to simply reflect dispersal from the oral cavity [17–22], we next thought to evaluate the contribution of the oral and nasal bacterial communities to the bronchial microbiota in our subjects. Although bronchial microbiota was found to be compositionally distinct from both the OW and NB, we hypothesized that a proportion of taxa detected in BB samples would also be detected in paired OW and NB samples from each individual. Indeed, we identified a number of specific OTUs that were shared with BB samples, significantly greater for OW samples (median/IQR 290/230–348; Fig. 5a) than for NB samples (median/IQR 26/13–48). Taxa also found in OW represented a large proportion (Fig. 5b; median/IQR 61/57–67%) of the total taxa detected in BB samples, a dramatically larger proportion than for the taxa shared between BB-NB samples (median/IQR 6/3–11%). Similarly, the phylogenetic diversity of the taxa shared between OW-BB (Additional file 1: Figure S7a; median/IQR 22/17–24) accounted for a larger proportion of the overall phylogenetic diversity of BB samples (Additional file 1: Figure S7b; median/IQR 66/63–76%), compared to the much smaller contribution to the diversity of taxa shared with NB (median/IQR 15/9–20%). Asthmatic subjects shared a greater number of OTUs between NB and BB samples than did HCs (Fig. 5c), but this was not the case for the number of OW-BB-shared OTUs, although the phylogenetic diversity of the NB-BB-shared taxa was similar for AAs and HCs (Mann-Whitney, *p* > 0.20). These results indicate that the oral cavity may serve as a source of bacteria for the lower airway microbiota in both healthy and asthmatic subjects, accounting for over 60% of the bronchial microbiota complexity. A novel observation of this study is that the nasal airway may also share microbiota members with the bronchial microbiota in adults and that in AA and ANA subjects more taxa are shared between the two compartments. Additionally, we show that OW and NB share distinct bacterial genera with the bronchial microbiota, since a comparison of the taxa shared between each of these niches and the bronchial microbiota was found to have no significant phylogenetic correlation (Mantel test; *r* = − 0.031, *p* = 0.69).

Of the known asthma-associated bacteria, a number were identified among those shared between BB and either or both nasal and oral compartments in AAs. As expected, the taxa shared between BB-OW samples included oral commensal genera such as *Fusobacterium*, *Porphyromonas*, and *Neisseria* (Fig. 5d), whereas *Streptococcus*, *Corynebacterium*, *Staphylococcus*, and to a lesser extent *Haemophilus* were among taxa detected with equal frequency as shared between BB-NB and BB-OW (Fig. 5d). It is important to note, however, that HCs also exhibited the presence of these genera among taxa shared between BB and either or both NB and OW samples (Additional file 1: Figure S7c). Taxa exclusively shared between bronchial and nasal microbiota included members of *Pseudomonas* and *Moraxella* in AAs (Fig. 5d). Importantly, *Moraxella* was not detected among the taxa shared between NB and BB of healthy subjects (Additional file 1: Figure S7c). Collectively, these findings indicate that the nasal taxa shared with bronchial airways in asthma may be few in number but great in importance, as they include bacterial genera previously associated with asthma in adults [1, 7] and asthma exacerbation in children [14–16].

### Nasal microbiota of asthmatics is associated with clinical and immunological features of allergic inflammation

**Fig. 6:**
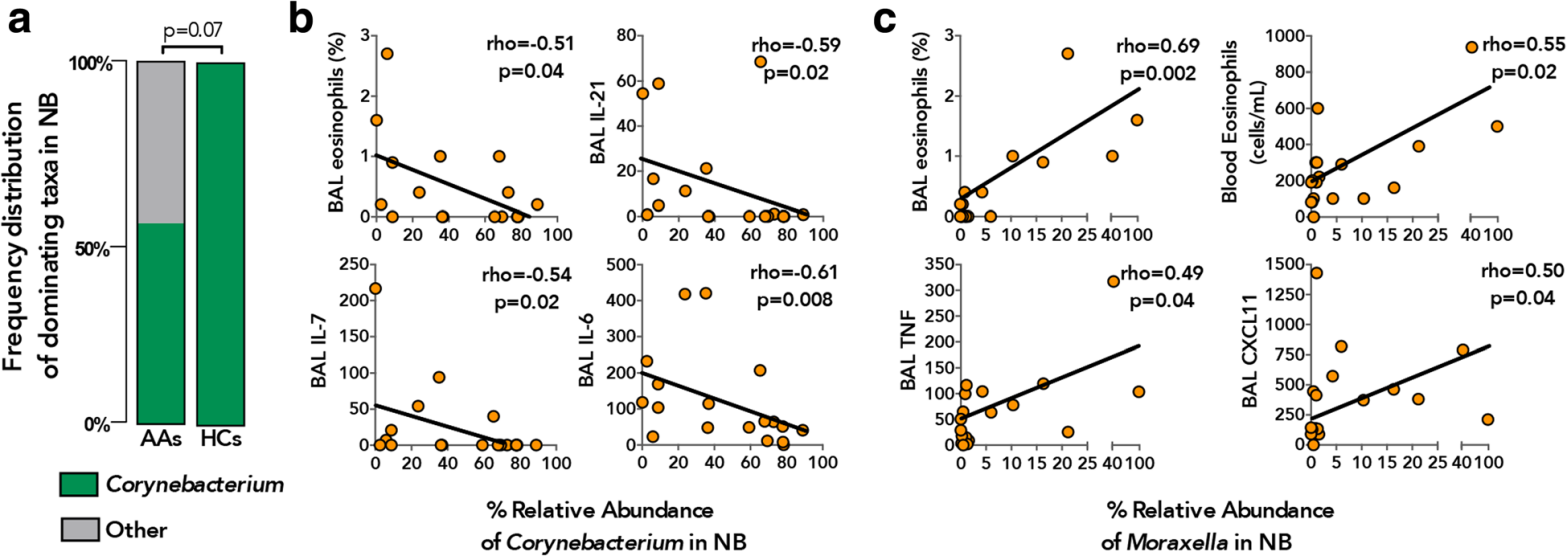
Nasal microbiota in asthmatic patients are associated with markers of systemic and bronchial inflammation. **a** Frequency distribution of *Corynebacterium* dominant communities in NB samples was lower in AAs (*n* = 18) compared to HC (*n* = 6) subjects (Fisher’s exact test). **b** The relative abundance of *Corynebacterium* in NB samples in AAs correlated negatively with a number of inflammatory markers of atopic asthma (Spearman correlation). **c** The relative abundance of *Moraxella* in NB samples in AAs correlated positively with a number of inflammatory markers of atopic asthma (Spearman correlation)

Recent studies of adult airway microbiota in respiratory disease have described specific patterns of bacterial colonization with distinct dominant genera in both the upper [23] and lower [24] airway compartments, differentially associated with divergent clinical outcomes. Accordingly, we assessed the distribution of bacterial genera dominating each of the paired samples and found that *Prevotella* was most frequently found to dominate BB and IS (Additional file 1: Figure S6c) and that there was no significant difference in its prevalence across these sample types (21/27 and 23/27, respectively; Fisher’s exact *p* = 0.73; Additional file 1: Figure S6c). In contrast, this genus dominated OWs with lower frequency (12/27) and did not dominate any of the NB samples (0/27; Fisher’s exact *p* = 0.024 and *p* < 0.0001, respectively; Additional file 1: Figure S6c). The microbiota of NBs also differed dramatically in the frequency with which *Corynebacterium* dominated these samples (19/27 Additional file 1: Figure S6c), further highlighting niche specificity in bacterial microbiota of the nasal airways. Our analysis of the frequency of *Prevotella* dominance in the four sample types from AAs and HC subjects showed them not to differ (Additional file 1: Figure S6d), and the consistency between BB and IS samples was observed in both groups. There was, however, a difference in the frequency of dominance by *Corynebacterium* in NB samples, which was found in all HCs but in only half of the AA subjects (Fig. 6a; Fisher’s exact *p* = 0.07). Possibly because of the small sample size and the mildness of the asthma of our subjects, we did not detect differences in the frequency distribution of other dominant genera between AAs and HC subjects, although we note that *Moraxella*-dominated communities in NB samples were detected only in asthmatic subjects (3/18 AAs, Additional file 1: Figure S6c).

In light of these observations, we enquired whether the relative abundance of the prevalent bacterial genera in the AAs was associated with a number of clinical and immunological features characteristic of the disease. Indeed, the relative abundance of *Actinomyces* in both BB and OW samples was inversely associated with serum IgE levels, as well as BAL neutrophil counts and G-CSF levels, respectively (Table 2). No significant associations were found between the inflammatory and clinical parameters examined and the relative abundance of the prevalent genera in IS samples, likely due to the complexity of the microbiota detected in this specimen type. Surprisingly, the most associations with features of atopic asthma was observed with bacterial genera detected in the nasal airways (Table 2) and included those previously implicated in asthma in pediatric studies [14]. For instance, the relative abundance of *Alloiococcus* positively correlated with PC_20_ (i.e., with lower bronchial reactivity) and that of *Corynebacterium* negatively correlated with a number of systemic and bronchial inflammatory markers including BAL eosinophil counts, IL-6, IL-7, and IL-21 levels (Table 2 and Fig. 6b). In contrast, the relative abundance of *Moraxella* and *Streptococcus* correlated positively with systemic and lower airway eosinophilia (Table 2 and Fig. 6c) and bronchial pro-inflammatory cytokines including TNF and IL-7, respectively. We did not detect a correlation between the relative abundance of these two genera (Spearman, *p* > 0.20), but both were negatively associated with the relative abundance of *Corynebacterium* (Spearman rho = − 0.51 and − 0.53, respectively, *p* = 0.030).

**Table 2:** Significant associations between the relative abundance of prevalent bacteria genera (present at ≥3% in either of the samples) and clinical and inflammatory parameters in AAs

Variables	rho^§^	*p* value	BH *p* value^&^
Relative abundance of *Actinomyces* in BB			
Serum IgE (IU/mL)	*− 0.57*	0.014	0.191
BAL neutrophils (%)	*− 0.63*	0.007	0.182
Relative abundance of *Actinomyces* in OW			
Serum IgE (IU/mL)	*− 0.64*	0.005	0.082
BAL GM-CSF	*− 0.63*	0.006	0.082
Relative abundance of *Alloiococcus* in NB			
PC_20_	*0.62*	0.006	0.164
Relative abundance of *Corynebacterium* in NB			
Blood eosinophils (cells/mL)	*− 0.49*	0.041	0.183
BAL eosinophils (%)	*− 0.51*	0.036	0.183
BAL IL-6	*− 0.61*	0.009	0.154
BAL IL-7	*− 0.54*	0.021	0.154
BAL IL-21	*− 0.56*	0.016	0.154
BAL MIP1-a	*− 0.47*	0.048	0.187
Relative abundance of *Moraxella* in NB			
Blood eosinophils (absolute)	*0.55*	0.017	0.152
Sputum eosinophils (%)	*0.64*	0.007	0.100
BAL eosinophils (%)	*0.69*	0.002	0.063
BAL ITAC	*0.50*	0.036	0.188
BAL TNF	*0.49*	0.042	0.188
Relative abundance of *Streptococcus* in NB			
Blood eosinophils (cells/mL)	*0.55*	0.017	0.198
BAL IL-7	*0.53*	0.022	0.198

^§^Analysis performed using Spearman correlation coefficient. ^&^*p* values were FDR corrected for multiple comparisons using Benjamini Hochberg method, with values ≤ 0.2 considered significant

Additionally, asthmatic subjects whose nasal microbiota was dominated by *Corynebacterium* exhibited significantly lower levels of pro-inflammatory BAL cytokines, specifically IL-6, IL-7, and IL-21 (Additional file 1: Table S5) compared to subjects exhibiting nasal microbiota dominated by other bacterial genera. Nasal microbiota of the *Corynebacterium*-dominated subgroup of asthmatic subjects were enriched for specific taxa belonging to *Corynebacterium*, *Streptococcus*, and *Lactobacillus* genera and were highly depleted for members of *Moraxella*, *Neisseriaceae*, *Streptococcus*, *Staphylococcus*, and *Haemophilus* compared to asthma subjects whose nasal microbiota was dominated by non-*Corynebacterium* genera (Additional file 1: Table S6). Whilst the bronchial microbiota in the same subjects was enriched for specific but unique members of *Microbacteriaceae*, *Lactobacillus*, and *Leptotrichia* and depleted for a number of taxa including *Streptococcus*, *Treponema*, *Fusobacterium*, and *Neisseria* (Additional file 1: Table S7). Thus, it appears that bronchial microbiota in asthmatics whose nasal microbiota is dominated by non-*Corynebacterium* bacterial genera are enriched for asthma-associated taxa, which may explain the elevated levels of pro-inflammatory cytokines in these subjects. However, apart from the observation that nasal *Corynebacterium* dominance of asthmatic subjects was more frequently observed in males (80%, Fisher’s exact *p* = 0.06; Additional file 1: Table S5), we were limited by a small sample size in this study to identify any other patient characteristics associated with this microbiological signature. Nevertheless, our observations suggest that predominant colonization of the nasal airways with members of *Corynebacterium* may serve as a biomarker for dampened inflammation associated with atopic asthma and are in agreement with prior observations of infant nasopharyngeal colonization with these genera being associated with lower risk for acute respiratory illness [14, 25], a risk factor for asthma development later in life [26–28]. Collectively, these findings suggest that the nasal microbiota in adult asthmatics, although distinct from bronchial bacterial communities, may prove useful biomarkers of bronchial and systemic inflammation associated with atopic asthma.

## Discussion

In this study, we aimed to address the compositional similarity between the bacterial microbiota detected in samples obtained by minimally invasive sampling approaches (nasal brushing, oral wash, sputum induction) to those detected in protected bronchial brushings, focusing on adults with mild, corticosteroid-naïve atopic asthma and adults without atopy or asthma. Our findings confirm niche specificity of microbiota in adult airways but indicate that the architecture of the bacterial communities in induced sputum, particularly in asthmatic subjects, resembles that in protected bronchial brushings more closely than do those in oral or nasal samples. Of the sampling methods examined, our observations collectively support the use of IS for inferring lower airway microbial community composition in asthmatic individuals, recognizing that IS microbiota may provide an incomplete picture of the bronchial microbiota, and inaccurately bias the microbiota composition to reflect oral taxa enrichment, especially in very mild airway disease, as in our cohort. However, our analyses by multiple approaches consistently found evidence in asthmatic subjects of greater similarity in IS and BB bacterial microbiota compared to non-asthmatic subjects. Further, while the bacterial communities found in the nasal mucosa were distinct from those in the bronchial mucosa, their composition was associated with markers of systemic and bronchial inflammation clinically relevant to asthma. Another novel observation we report in this study is that nasal airways are a potential source of distinct bacterial taxa for lower airways in adults, and that this inoculum is richer in atopic asthmatic subjects.

Although our findings provide evidence in support of using less-invasive sampling methods to study the airway microbiota in larger numbers of asthmatic patients, important limitations of these findings must be acknowledged. The number of subjects from our cohort who had all paired sample types available for analysis was relatively small, especially a paucity of NB samples from atopic subjects without asthma, an important control group to compare against subjects with atopic asthma. Thus, the observation of a richer shared microbiota composition between NB and BB samples in asthmatic than in healthy subjects could reflect associations with atopic status and immune responses. Also, a relatively constricted scope of asthma phenotype was captured in our cohort who all had mild, corticosteroid-naïve atopic asthma. The similarities and dissimilarities we quantified between upper airway and bronchial samples in bacterial microbiota composition may or may not hold true in other asthma phenotypes or with worsened disease activity. Our analysis also focused only on adult subjects, and it is possible that findings may differ in infants and young children due to differences in anatomical distance between upper and lower airway compartments. A major strength of this study is the standardized collection and analysis of paired upper and lower airway sample types, all of which were collected either on the same day (NB, OW and BB at bronchoscopy visit) or within 2 weeks (IS) prior to the bronchoscopy.

Our findings confirm that different compartments of the airways harbor compositionally distinct microbiota in patients with asthma, as they do in healthy adults [17]. They further reveal the bacterial communities colonizing the nasal cavity to be significantly scarcer than those colonizing the lower airways. That we should find the microbial composition of induced sputum to differ overall from bronchial brush is not surprising, given that an earlier study reported distinct differences in the microbial communities detected in BAL and brush obtained from the same subjects [1]. We expected comparison of the induced sputum and protected bronchial brushes collected in our study to reveal dissimilarities, considering the unavoidable contamination of bronchial secretions by saliva during the process of sputum collection, as well as the difference in spatial topography of the airways sampled by either method. Indeed, we found the microbiota of induced sputum to be compositionally most similar to oral wash but still to resemble the bronchial brush microbiota more closely than did oral wash or nasal brush samples. The taxonomic architecture of induced sputum samples resembled that of the bronchial brush samples, particularly in atopic subjects, and the taxa shared between the two sample types included those previously reported to be associated with asthma, including members of *Haemophilus*, *Fusobacterium*, and *Neisseria* [1, 3–5, 7]. These observations suggest that the bacterial communities in induced sputum, although distinct from those of the bronchial mucosa, capture useful aspects of the latter in asthma. This finding is encouraging for future mechanism-oriented studies aimed at elucidating the contribution of the airway microbiome to asthma heterogeneity, enabling expansion of studies to include larger cohorts of patients and the ability to study patients repeatedly over time.

Nasal colonization with *S. aureus has* previously been reported as a risk factor for asthma diagnosis in children and young adults [29], while *Staphylococcus*-derived products have been observed to directly induce pro-inflammatory Th2 inflammation in human nasal epithelium [30] and exacerbate allergic airway inflammation in experimental model of asthma in mice [31]. Our observation of enrichment of *Staphylococcus* in the nasal microbiota of atopic asthmatics in our cohort further supports a possible role for this pathobiont in asthma. Further, despite our finding that nasal and bronchial microbiota are compositionally distinct in asthmatic as well as healthy [17] adults, we identified a suite of bacterial taxa shared between these two compartments that accounted for over 15% of the bronchial phylogenetic diversity in our atopic asthma subjects. Although this contribution of the nasal compartment is dwarfed by the contribution of taxa from the oral cavity, both compartments contribute distinct and important asthma-associated taxa to the lower airways in adult asthma, as has been shown in children with chronic lung disease [32]. In particular, we show that the relative abundance of *Moraxella* correlates positively between paired nasal and bronchial brush samples, and members of this genus are among the taxa shared between the two compartments in some of our asthmatic but in none of the healthy subjects. Intriguingly, the relative abundance of nasal *Moraxella* was positively associated with systemic and airway eosinophil counts, suggesting associations with allergic inflammation, as well as with asthma-associated [33, 34] pro-inflammatory chemokine CXCL11 and TNF levels in bronchial lavage fluid. Increased expression of nasal epithelial TNF has also been reported in children and adolescents with asthma whose nasal microbiome was enriched for *M. catarrhalis* [35].

This association of nasal *Moraxella* with inflammatory cells and cytokines in our asthmatic subjects contrasted with our findings related to detection of *Corynebacterium* in nasal brushings. *Corynebacterium* was detected as a dominant genus in the nasal samples from all healthy subjects, but in only half of our asthmatic subjects, in whom the abundance of this genus was negatively associated with the presence of *Moraxella* and *Streptococcus* in nasal brushes and of eosinophils in the blood and of asthma-associated [30, 33] inflammatory cytokines in BAL fluid. Previous findings from a number of pediatric studies have shown differences in nasal microbiota to be related to differences in asthma-related outcomes [12–16, 25], suggesting that the microbiota of the nasal airways may be a biomarker of asthma risk in children. Of particular interest in this context is the finding of an inverse relationship in the abundance of *Corynebacterum* and both *Moraxella* [25, 36] and *Streptococcus* [36–38] in the nasal microbiota of young children and inverse association with respiratory health [14, 25], which appear to be sustained in adult airways of asthmatic subjects. The mechanisms driving these antagonistic interactions may involve commensal bacteria directly inhibiting outgrowth of a pathobiont, as was shown for commensal *Corynebacterium’s* inhibition of *S. pneumoniae* growth in- vitro [38], but much more work is needed to explore these relationships and their subsequent associations with host immune function. At the least, our findings suggest that additional studies of the nasal microbiota associations to asthma-related inflammation, phenotype, clinical course, or response to therapy are warranted.

## Conclusion

We conclude that in adults, induced sputum is superior to nasal brush or oral wash for studying bronchial microbiota in asthma. Although compositionally similar, the microbiota in induced sputum are distinct from bronchial microbiota in reflecting enrichment of oral bacterial taxa. Although the nasal microbiota was highly distinct from that of the oral or bronchial compartments, the abundance of specific bacterial genera in nasal brushes from asthmatic subjects (*Corynebacerium* and *Moraxella)* was associated with abundance or depletion in bronchial brushes of genera previously reported as associated with asthma, and further to be associated with markers of systemic and bronchial inflammation. Future studies designed to elucidate bacterial mechanisms contributing to the pathophysiology of asthmatic airway inflammation may therefore benefit from parallel sampling of lower and upper airway compartments.

## Methods

### Study population and sample collection

The study cohort consisted of a subset of subjects enrolled in the NHLBI AsthmaNet microbiome study NCT01537133 [3] and included 22 atopic asthmatics (AA), 12 atopic non-asthmatics (ANA) and 11 non-atopic healthy control subjects (HC) whose characteristics are summarized in Table 1 and Additional file 1: Table S1. Atopy was defined by serologic evidence (> 0.35 kU/l) of sensitivity to ≥ 1 of 12 aeroallergens (specific IgE by ImmunoCap; Thermo-Scientific). Asthma was confirmed by measurement of airway responsiveness (methacholine PC_20_ ≤ 8 mg/mL or FEV_1_ improvement ≥ 12% post-albuterol). Allergic inflammation in asthmatic subjects was assessed by measurement of circulating and sputum inflammatory cells and BAL levels of inflammatory cytokines [33]. At enrollment, asthmatics had been clinically stable for 3 months, without the use of a controller medication in the preceding 6 months. Exclusion criteria included a history of smoking, respiratory infection within 6 weeks or antibiotic use within 3 months of enrollment. Each subject signed informed consent approved by their center’s IRB; an NHLBI-appointed Data Safety Monitoring Board (DSMB) oversaw the study conduct.

Samples processed for microbiota analysis were collected across nine clinical centers and included 45 paired protected bronchial brushings (BB), oral wash (OW) and induced sputum (IS) samples. In subset of 27 of these 45 subjects, intranasal brushings (NB) were also collected (Additional file 1: Table S2). The distribution of samples did not vary significantly across clinical centers (Chi-square; *p* > 0.10). IS samples were collected as previously described [39] within a 2-week window (median 8 [IQR 8–10] days) from the date of bronchoscopy and those with > 80% squamous epithelial cell counts were excluded from the study. All samples intended for microbial analysis were stored in RNAprotect Saliva Reagent (QIAgen) at − 80 **°**C until processing.

### BAL cytokine multiplex testing

Bronchoalveolar lavage (BAL) supernatants collected during bronchoscopy visit were concentrated by applying 4 mL of sample to Amicon Ultra-4 Centrifugal Filter Unit tubes (Millipore) following centrifugation at 4000*g* for 40 min at 4 **°**C. Concentrated BAL fluid was assayed using a MILLIPLEX MAP human high sensitivity T cell panel 21-plex immunology multiplex assay (Millipore) with antibody-coated beads for detection of Fractalkine, TNF, MIP-3α, MIP-1β, MIP-1α, ITAC, IL-23, IL-21, IL-17A, IL-13, IL-12 (p70), IL-10, IL-8, IL-7, IL-6, IL-5, IL-4, IL-2, IL-1β, IFNγ, and GM-CSF. Standards and experimental samples were tested in duplicate. Results were acquired on a Labscan 200 analyzer (Luminex) using Bio-Plex manager software 6.1 (Bio-Rad). A 5-point logistic regression curve was used to calculate the concentration from the fluorescence intensity of the bead measurements. Samples that were below the level of detection were assigned one-half the lowest detectable value for that analyte. Out of the 21 targeted cytokines, only 11 had detectable levels in at least 4 (i.e., 20%) subjects; these were included in the comparisons with the microbiological data.

### Nucleic acid extraction and 16S rRNA-based sequencing

Nucleic acids were extracted as previously described [7] using a modified bead-beating protocol and the AllPrep kit (QIAgen). The variable region 4 (V4) of the 16S rRNA gene was amplified using 515F/806R primer combination as previously described [40, 41]. Amplicons were purified using SPRI beads (Beckman Coulter) or in the presence of multiple bands, gel-extracted with a QIAgen Gel Extraction kit (QIAgen), analyzed on Bioanalyzer (Aligent), and quantified using the Qubit HS dsDNA kit (Invitrogen). Only samples with amplicons ≥ 10 ng were sequenced. Blank control DNA extracts were also amplified, bead purified, and sequenced. Samples with sufficient amplicon were pooled at 50 ng per sample, with a blank control per 30–35 samples. The barcoded, pooled library was quantified using the Qubit HS dsDNA kit (Invitrogen), denatured and 5pM was loaded onto the Illumina MiSeq cartridge (V3) in combination with a 15% (*v*/*v*) of denatured 12.5pM PhiX for sequencing.

### Sequence data processing and quality control

Paired-end sequences were combined using FLASh version 1.2.7 [42]. Sequence analysis was performed using the Quantitative Insights into Microbial Ecology (QIIME) pipeline [43]. Raw sequences were de-multiplexed by barcode and quality filtered by removing low-quality sequences. Sequences with three or more consecutive bases with a Q score < 30 were truncated and discarded if the length was less than 75% of the original 250 bp read length. Sequences were aligned using PyNAST [44], and operational taxonomic units (OTUs) were picked at 97% sequence identity using UCLUST [45] against the Greengenes database [46]. Reads that failed to hit the reference sequence collection were retained and clustered de novo. PyNAST-aligned sequences were chimera checked using ChimeraSlayer and putative chimeras as well as OTUs identified in negative controls (Additional file 1: Table S8) were removed from the working OTU table. A phylogenetic tree was built using FastTree [47] and used to compute Faith’s Phylogenetic Diversity and UniFrac distances on OTU table multiply rarefied [48] to 26,185 sequences per sample.

### Statistical analysis

All statistical analyses were performed as indicated in QIIME, R environment or using PRISM software. Statistical tests used were selected based on the outcome of Shapiro-Wilk’s normality test coupled with a graphical approach. Wilcoxon matched-pairs rank test was used for comparison between correlated sample types. Welch’s corrected *t* test, or Mann-Whitney test and Chi-square or Fisher’s exact test where appropriate were used to determine significant differences in comparisons between independent study groups. Spearman correlation was used to test for an association between the relative abundance of bacterial genera in paired sample types and immunological and clinical parameters in AA subjects. Ordination was visualized using principal coordinate analysis (PCoA) on unweighted UniFrac distance matrix and plotted using Emperor [49]. Significant differences in beta-diversity between paired samples were calculated on unweighted UniFrac PC1 coordinates (as a response variable) using Linear mixed effects (LME) model ([50] using *lmerTest* package in R). Three-model approach (Poisson, negative binomial, and zero-inflated negative binomial mixed-effect models) corrected [51] for multiple testing (*q* < 0.10) as previously described [52] was used to determine specific OTUs in NBs or BB which differed in relative abundance between groups. Mantel test [53] based on unweighted UniFrac distance was used to compare phylogenetic contribution of nasal and oral compartments to BB microbiota based on OTUs shared between paired samples.

## Additional file

Additional file 1: Supplemental information includes **Figures S1-7** and **Tables S1-8.** (DOCX 10754 kb)

## Abbreviations

AA: Atopic adults with mild asthma
ANA: Atopic non-asthmatic adults
BAL: Broncho-alveolar lavage
BB: Protected bronchial brushings
HC: Healthy controls
IS: Induced sputum
LEM: Linear mixed effects model
NB: Nasal brushings
OTU: Operational taxonomic unit
OW: Oral wash
