# Supplementary material for: Bacterial biogeography of adult airways in atopic asthma
Source: Microbiome. 2018 Jun 9;6:104. doi: 10.1186/s40168-018-0487-3 (PMC5994066; doi:10.1186/s40168-018-0487-3)
Supplement: Supplementary file 1 — Supplemental information includes Figures S1-7 and Tables S1-8. (DOCX 10754 kb) [file 40168_2018_487_MOESM1_ESM.docx]

**Fig. S1:** Alpha diversity in the microbiota of different specimen types demonstrating that the IS harbors significantly more diverse bacterial communities than the BB or the OW. **a.** Bacterial richness as indicated by the total number of taxa detected in each sample type. **b.** Shannon index of bacterial diversity in each sample type. **c.** Phylogenetic Faith’s index of bacterial diversity in each sample type. **d.** Pielou’s index of bacterial evenness in each sample type. Statistical significance was determined using Wilcoxon matched-pairs signed rank test; p>0.05 are not shown.

**Fig. S2:** **a.** While the relationship between sample types was similar in AAs and HCs, Faith’s Phylogenetic diversity trended to be higher in AAs compared to HCs in BB samples (n=24; ^$^Welch’s t-test). **b.** Faith’s Phylogenetic diversity trended to be higher in AAs compared to HC subjects in BB samples when all available sample pairs were compared (n=45; ^$^Welch’s t-test). Differences in diversity between sample types remained conserved in AAs, ANAs and HC subjects (Wilcoxon matched-pairs signed rank test).


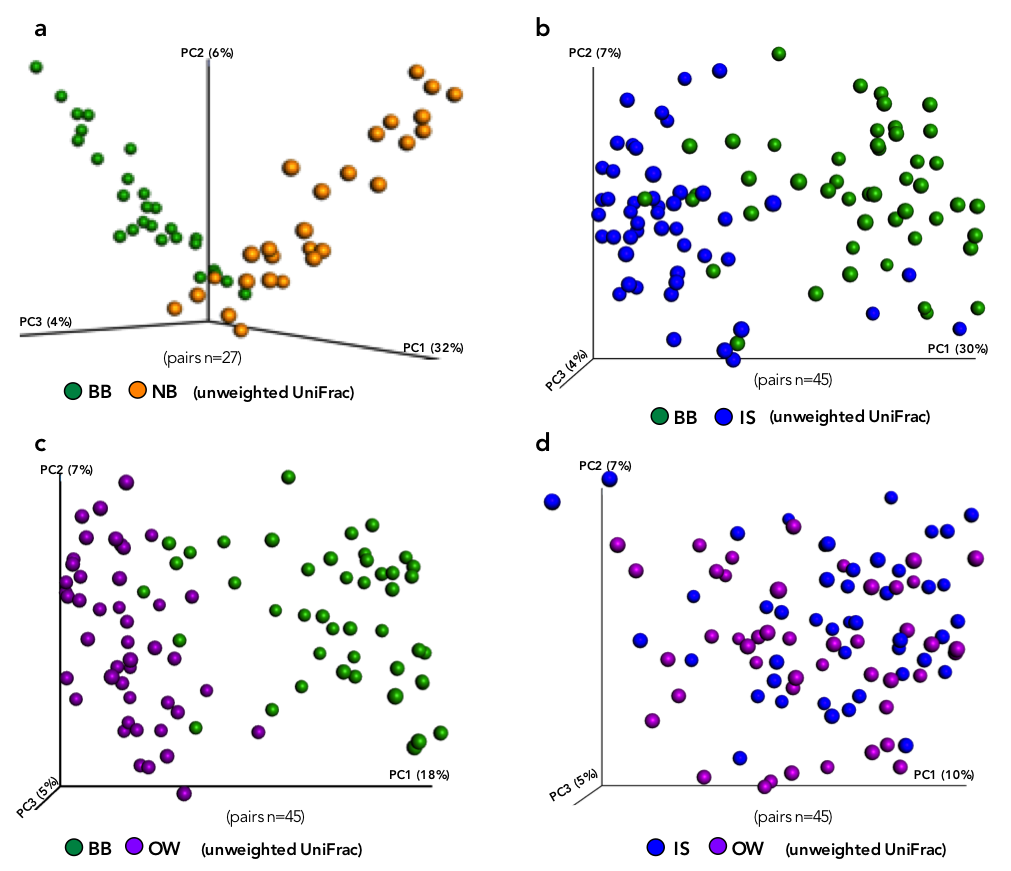


**Figure S3: a.** Principal coordinates (PCoA) analysis between paired BB and NB samples (n=27) shows compositional dissimilarity (LME 𝛃=-0.55, p<0.001). **b.** PCoA analysis between paired BB and IS samples (n=45) shows compositional dissimilarity (LME 𝛃=-0.27, p<0.001). **c.** PCoA analysis between paired BB and OW samples (n=45) shows compositional dissimilarity (LME 𝛃=-0.31, p<0.001). **d.** PCoA analysis between paired IS and OW samples (n=45) shows compositional dissimilarity (LME 𝛃=-0.04, p=0.019), though less so than the other comparisons.


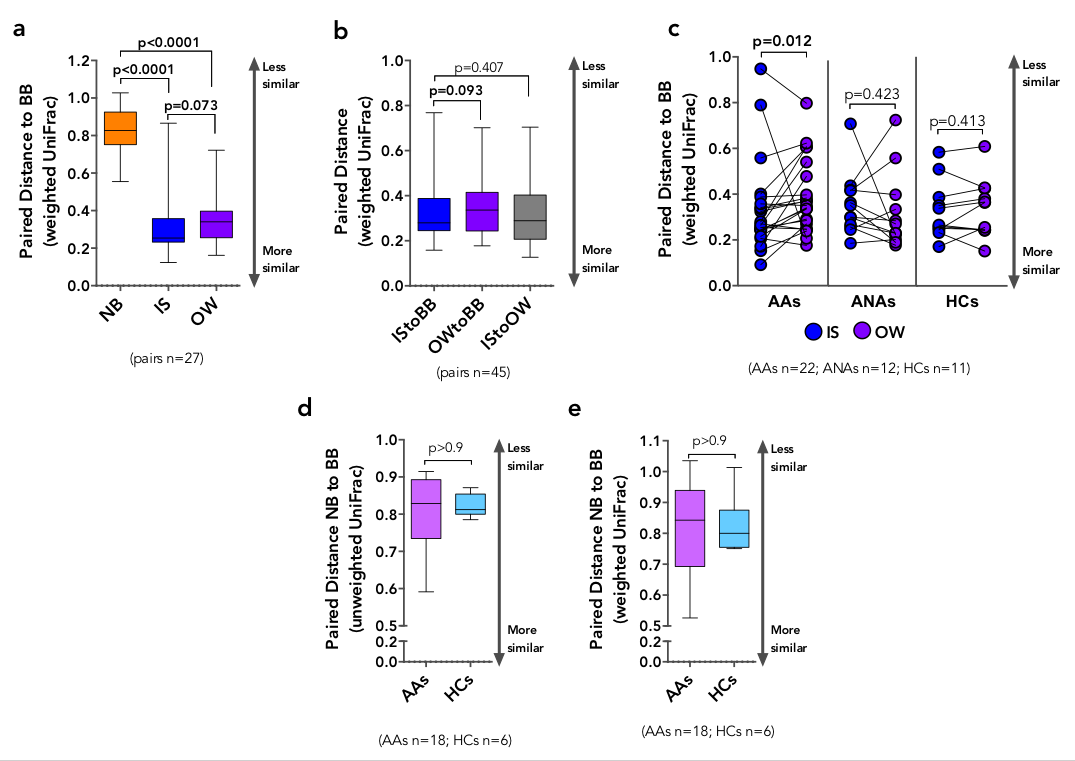


**Fig. S4:** **a.** Mean intra-subject paired distance to BB shows that the phylogenetic communities that comprise NB microbiota are most distinct from BB (weighted UniFrac; Wilcoxon matched-pairs signed rank test; Whiskers extend to 95% confidence interval). **b.** Relatively sorter mean intra-subject paired distance between IS-BB compared to OW-BB, suggests that the IS microbiota are more representative of BB than OW is of BB (weighted UniFrac; Wilcoxon matched-pairs signed rank test; Whiskers extend to 95% confidence interval). **c.** Shorter mean intra-subject paired distance is observed between BB and IS compared to OW in AAs but not ANAs nor HCs (Wilcoxon matched-pairs signed rank test). **d.** Mean intra-subject unweighted UniFrac paired distance between NB and BB in AAs and HC subjects (Mann Whitney test; Whiskers extend to 95% confidence interval). **e.** Mean intra-subject weighted UniFrac paired distance between NB and BB in AAs and HC subjects (Mann Whitney test; Whiskers extend to 95% confidence interval).


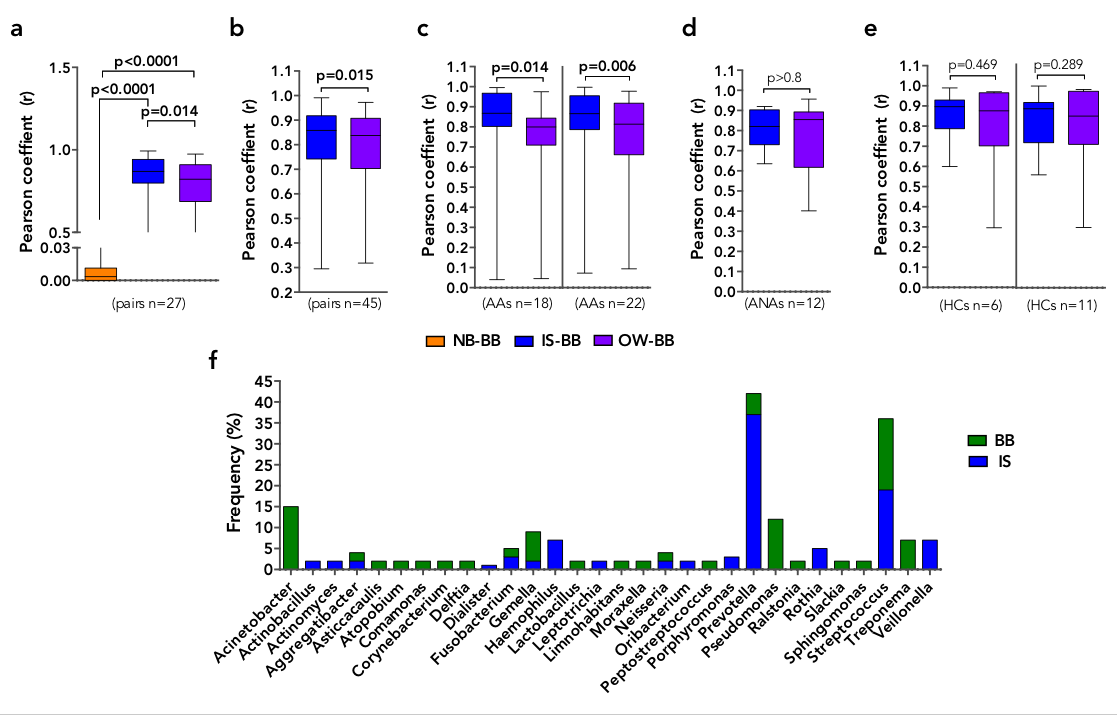


**Fig. S5:** **a.** Mean Person coefficient for the overall relative abundance of bacterial genera detected in 27 BB and other paired sample types (Wilcoxon matched-pairs signed rank test; Whiskers extend to 95% confidence interval). **b.** Mean Person coefficient for the overall relative abundance of bacterial genera detected in 45 BB and other paired sample types (Wilcoxon matched-pairs signed rank test; Whiskers extend to 95% confidence interval). **c.** Mean Person coefficient for the overall relative abundance of bacterial genera detected in BB and IS or OW samples from AA subjects (Wilcoxon matched-pairs signed rank test; Whiskers extend to 95% confidence interval). **d.** Mean Person coefficient for the overall relative abundance of bacterial genera detected in BB and IS or OW samples from ANA subjects (Wilcoxon matched-pairs signed rank test; Whiskers extend to 95% confidence interval). **e.** Mean Person coefficient for the overall relative abundance of bacterial genera detected in BB and IS or OW samples from HC subjects (Wilcoxon matched-pairs signed rank test; Whiskers extend to 95% confidence interval). **f.** Frequency distribution of bacterial genera (present in at least 20% of each sample type) for OTUs detected exclusively in IS or BB samples.

**Fig. S6:** **a.** Asthmatic subjects trended to have a higher relative abundance of *Staphylococcus* in NB samples compared to HCs (Mann-Whitney test). **b.** Asthmatic subjects showed significant enrichment in a number of specific *Staphylococcus* taxa in NB samples compared to HCs. Significance was determined using NB/ZINB regression models and corrected for false discovery, q<0.1. Length of bar represents the difference in mean relative abundance between groups, log_2_ transformed. Only OTUs enriched ≥2 fold are presented. **c.** Frequency of dominant bacterial genera distribution in paired samples (n=27). **d.** Frequency of *Prevotella* dominated communities in the four sample types was similar in AA and HC subjects.

**Fig. S7:** **a.** Faith’s phylogenetic diversity (PD) of taxa shared with BB samples is higher for OW compared to NB. **b.** PD of taxa shared represents a larger proportion of the overall diversity detected in BB samples for paired OW compared to NB. **c.** Distribution of specific taxa shared with paired BB is distinct between NB and OW samples in HCs. (Genera identified in at least 20% of subjects in the same sample type; Statistical significance between OW and NB genera was determined using Fisher’s exact test where ***p≤0.01, **p≤0.05, *p≤0.10).

**Table S1** Clinical characteristics for a subset of 27 subjects for whom nasal brushes were collected.

| Variable | **Allergic asthmatics**  **(AA)**  (n = 18) | **Non-allergic non-asthmatics**  **(ANA)**  (n = 6) | **p-value**^#^ |
| --- | --- | --- | --- |
| Age (yrs) | 39 (27 - 45) | 38 (25 - 49) | NS |
| ACQ Score (Baseline)* | 0.7 (0.3 - 1.0) | - | **-** |
| % Male | 56% | 50% | NS^€^ |
| % White | 67% | 67% | NS^€^ |
| BMI (kg/m^2^) | 27 (23 - 30) | 27 (25 - 28) | NS |
| FEV1 % predicted pre- Alb^¥^ | 79 (69 - 96) | 105 (95 - 117) | **0.003** |
| FEV1 % predicted post- Alb^¥^ | 95 (82 - 105) | 111 (98 - 121) | **0.023** |
| Change in FEV% | 10.5 (6.0 - 15.0) | 4.5 (2.3 - 5.8) | **0.002** |
| PC20 (methacholine) | 1.4 (0.5 - 3.7) | >32^$^ | **<0.0001** |
| Serum IgE (EU/mL)^¢^ | 195.0 (54.3 - 313.0) | 18.5 (4.3 - 39.0) | **0.001** |
| Allergic rhinitis (%) | 50% | 0% | **0.051**^€^ |
| Blood neutrophils (%) | 53.1 (48.0 - 57.7) | 59.0 (53.1 - 68.0) | *0.075* |
| Blood eosinophils (%) | 3.9 (2.0 - 5.3) | 2.5 (1.0 - 3.1) | *0.068* |
| Sputum neutrophils (%) | 53.8 (31.0 - 64.7) | 41.8 (36.8 - 59.9) | NS |
| Sputum eosinophils (%) | 0.7 (0.2 - 2.7) | 0.1 (0.0 - 0.4) | **0.044** |
| BAL GM-CSF (pg/mL) | 319 (187 - 579) | 157 (101 - 245) | *0.066* |
| BAL IL6 (pg/mL) | 65 (37 - 179) | 93 (60 - 148) | NS |
| BAL IL7 (pg/mL) | 0.3 (0.3 - 25.7) | 0.3 (0.3 - 2.2) | NS |
| BAL IL8 (pg/mL) | 1271 (435 - 2213) | 1011 (534 - 1695) | NS |
| BAL CXCL11 (pg/mL) | 293 (123 - 491) | 359 (169 - 567) | NS |
| BAL MIP-1α (pg/mL) | 73 (27 - 139) | 100 (40 - 134) | NS |
| BAL MIP-1β (pg/mL) | 287 (109 - 422) | 247 (125 - 521) | NS |
| BAL MIP-3α (pg/mL) | 384 (162 - 782) | 246 (174 - 581) | NS |
| BAL TNF (pg/mL) | 57 (14 - 104) | 66 (28 - 100) | NS |
| BAL IL-1β (pg/mL) | 0.04 (0.04 - 4.2) | 0.04 (0.04 - 0.04) | NS |
| BAL IL21 (pg/mL) | 0.8 (0.04 - 17.8) | 0.04 (0.04 - 5.8) | NS |

All values are medians (IQR).*ACQ, Asthma Control Questionnaire. ^¥^Alb-Albuterol ^$^Methacholine challenge was stopped at 32 mg/dL and PC_20_ for these subjects was censored. ^¢^Number of positive specific IgE (sIgE >0.35 kU/l) from a total of 12 aeroallergens tested by ImmunoCap assay. Statistical significance was determined using ^#^Mann-Whitney or ^€^ Fisher’s exact test with p-values >0.1 assigned NS.

**Table S2** Breakdown of paired samples by group included in the current study.

|  | AAs | ANAs | HC | Total # samples processed |
| --- | --- | --- | --- | --- |
| # samples- paired NB-BB-IS-OW (n=27) | 18 | 3 | 6 | 108 |
| # samples- paired BB-IS-OW (n=45) | 22 | 12 | 11 | 135 |

**Table S3** Correlation between the overall relative abundance of bacterial genera detected in 27 BB and other paired sample types.

| BB | **NB r^#^** | **95%CI**^&^ | **p**^§^ | **IS r^#^** | **95%CI**^&^ | **p**^§^ | **OW r^#^** | **95%CI**^&^ | **p**^§^ |
| --- | --- | --- | --- | --- | --- | --- | --- | --- | --- |
| 1 | **0.011** | [-0.072, 0.094] | 1 | **0.798** | [0.766, 0.827] | 0.027 | **0.862** | [0.839, 0.882] | 0.027 |
| 2 | **-0.002** | [-0.085, 0.081] | 1 | **0.996** | [0.995, 0.996] | 0.027 | **0.975** | [0.971, 0.979] | 0.027 |
| 3 | **-0.004** | [-0.087, 0.079] | 1 | **0.841** | [0.814, 0.863] | 0.027 | **0.822** | [0.793, 0.847] | 0.027 |
| 4 | **-0.007** | [-0.089, 0.076] | 1 | **0.942** | [0.932, 0.951] | 0.027 | **0.973** | [0.968, 0.977] | 0.027 |
| 5 | **-0.004** | [-0.086, 0.079] | 1 | **0.896** | [0.878, 0.911] | 0.027 | **0.910** | [0.894, 0.923] | 0.027 |
| 6 | **0.004** | [-0.079, 0.086] | 1 | **0.681** | [0.634, 0.723] | 0.027 | **0.687** | [0.641, 0.728] | 0.027 |
| 7 | **-0.002** | [-0.084, 0.081] | 1 | **0.044** | [-0.039, 0.127] | 0.324 | **0.045** | [-0.038, 0.127] | 0.216 |
| 8 | **0.066** | [-0.017, 0.148] | 1 | **0.741** | [0.701, 0.776] | 0.027 | **0.734** | [0.693, 0.770] | 0.027 |
| 9 | **0.055** | [-0.028, 0.137] | 0.243 | **0.862** | [0.839, 0.882] | 0.027 | **0.838** | [0.812, 0.861] | 0.027 |
| 10 | **-0.004** | [-0.087, 0.079] | 1 | **0.966** | [0.960, 0.971] | 0.027 | **0.838** | [0.812, 0.861] | 0.027 |
| 11 | **-0.001** | [-0.083, 0.082] | 1 | **0.982** | [0.979, 0.985] | 0.027 | **0.952** | [0.944, 0.959] | 0.027 |
| 12 | **0.007** | [-0.076, 0.090] | 1 | **0.920** | [0.907, 0.932] | 0.027 | **0.825** | [0.797, 0.850] | 0.027 |
| 13 | **0.012** | [-0.071, 0.095] | 1 | **0.909** | [0.894, 0.923] | 0.027 | **0.842** | [0.816, 0.865] | 0.027 |
| 14 | **0.008** | [-0.075, 0.091] | 1 | **0.850** | [0.825, 0.872] | 0.027 | **0.837** | [0.811, 0.861] | 0.027 |
| 15 | **-0.003** | [-0.086, 0.080] | 1 | **0.865** | [0.843, 0.885] | 0.027 | **0.395** | [0.323, 0.463] | 0.081 |
| 16 | **-0.004** | [-0.087, 0.079] | 1 | **0.040** | [-0.043, 0.122] | 0.567 | **0.681** | [0.634, 0.723] | 0.027 |
| 17 | **0.010** | [-0.073, 0.092] | 1 | **0.909** | [0.893, 0.922] | 0.027 | **0.936** | [0.925, 0.946] | 0.027 |
| 18 | **0.010** | [-0.073, 0.093] | 1 | **0.900** | [0.883, 0.915] | 0.027 | **0.799** | [0.767, 0.827] | 0.027 |
| 19 | **0.001** | [-0.082, 0.084] | 1 | **0.990** | [0.988, 0.991] | 0.027 | **0.970** | [0.965, 0.975] | 0.027 |
| 20 | **0.004** | [-0.079, 0.086] | 1 | **0.972** | [0.967, 0.976] | 0.027 | **0.663** | [0.614, 0.707] | 0.054 |
| 21 | **0.361** | [0.287, 0.431] | 0.054 | **0.983** | [0.980, 0.986] | 0.027 | **0.719** | [0.676, 0.757] | 0.027 |
| 22 | **0.002** | [-0.081, 0.085] | 1 | **0.804** | [0.773, 0.831] | 0.027 | **0.782** | [0.748, 0.812] | 0.027 |
| 23 | **0.009** | [-0.074, 0.092] | 1 | **0.870** | [0.848, 0.889] | 0.027 | **0.801** | [0.769, 0.829] | 0.027 |
| 24 | **0.096** | [0.013, 0.177] | 0.999 | **0.852** | [0.828, 0.873] | 0.027 | **0.725** | [0.683, 0.762] | 0.027 |
| 25 | **0.048** | [-0.035, 0.131] | 0.999 | **0.897** | [0.879, 0.912] | 0.027 | **0.964** | [0.958, 0.969] | 0.027 |
| 26 | **-0.005** | [-0.087, 0.078] | 1 | **0.600** | [0.544, 0.650] | 0.027 | **0.296** | [0.219, 0.370] | 0.108 |
| 27 | **-0.001** | [-0.084, 0.082] | 1 | **0.777** | [0.742, 0.808] | 0.027 | **0.590** | [0.533, 0.642] | 0.054 |

^#^Analysis performed on bacterial genera detected in 27 paired BB, NB, IS and OW samples using Pearson correlation. ^&^The confidence intervals were constructed at a confidence level of 95.0% using Fisher's z-transformation. ^§^The nonparametric Bonferroni-corrected p-values were calculated using a two-sided permutation test with 999 permutations.

**Table S4** Relationship between the relative abundance for prevalent bacterial genera (present at ≥3% in any one of the samples) detected in BB and other sample types.

| Genera^&^ | Sample Comparison^§^ | rho | p-value |
| --- | --- | --- | --- |
| *Actinomyces* | *BB and NB* | -0.327 | 0.096 |
|  | *BB and IS* | 0.219 | 0.272 |
|  | *BB and OW* | 0.279 | 0.159 |
|  | *IS and OW* | **0.479** | **0.011** |
| *Corynebacterium* | *BB and NB* | -0.170 | 0.401 |
|  | *BB and IS* | **0.440** | **0.023** |
|  | *BB and OW* | **0.680** | **< 0.001** |
|  | *IS and OW* | **0.478** | **0.012** |
| *Porphyromonas* | *BB and NB* | 0.210 | 0.297 |
|  | *BB and IS* | **0.690** | **< 0.001** |
|  | *BB and OW* | **0.440** | **0.020** |
|  | *IS and OW* | **0.628** | **0.0005** |
| *Prevotella* | *BB and NB* | -0.150 | 0.454 |
|  | *BB and IS* | **0.382** | **0.049** |
|  | *BB and OW* | 0.228 | 0.252 |
|  | *IS and OW* | **0.566** | **0.002** |
| *Staphylococcus* | *BB and NB* | -0.028 | 0.888 |
|  | *BB and IS* | 0.276 | 0.164 |
|  | *BB and OW* | 0.159 | 0.429 |
|  | *IS and OW* | **0.593** | **0.001** |
| *Alloiococcus* | *BB and NB* | 0.207 | 0.300 |
|  | *BB and IS* | 0.291 | 0.141 |
|  | *BB and OW* | NA | NA |
|  | *IS and OW* | NA | NA |
| *Streptococcus* | *BB and NB* | 0.124 | 0.539 |
|  | *BB and IS* | **0.550** | **0.003** |
|  | *BB and OW* | **0.680** | **< 0.001** |
|  | *IS and OW* | **0.680** | **< 0.001** |
| *Leptotrichia* | *BB and NB* | 0.081 | 0.686 |
|  | *BB and IS* | **0.540** | **0.003** |
|  | *BB and OW* | **0.720** | **< 0.001** |
|  | *IS and OW* | **0.507** | **0.007** |
| *Moraxella* | *BB and NB* | **0.506** | **0.007** |
|  | *BB and IS* | 0.137 | 0.495 |
|  | *BB and OW* | 0.238 | 0.232 |
|  | *IS and OW* | 0.238 | 0.232 |

^§^Analysis performed between 27 paired BB, NB, IS and OW samples using Spearman correlation coefficient. ^&^For relative abundance distribution of these genera see Fig.3.

**Table S5** Clinical characteristics of asthmatic (AA) patients based on the dominant bacterial genera detected in their nasal microbiota.

| **Variable** | **AA *Corynebacterium***  (n = 10) | **AA**  **non-*Corynebacterium***  (n = 8) | **p-value**^#^ |
| --- | --- | --- | --- |
| Age (yrs) | 40 (29 - 45) | 38 (25 - 47) | NS |
| ACQ Score (Baseline)* | 0.5 (0.3 - 0.9) | 0.7 (0.3 - 1.1) | NS |
| % Male | 80% | 25% | *0.054*^€^ |
| % White | 60% | 75% | NS^€^ |
| BMI (kg/m^2^) | 28 (24 - 30) | 24 (23 - 32) | NS |
| FEV1 % predicted pre-Alb^¥^ | 79 (69 - 97) | 78 (69 - 94) | NS |
| FEV1 % predicted post-Alb^¥^ | 89 (82 - 106) | 97 (81 - 104) | NS |
| Change in FEV% | 7.5 (5.0 - 12.8) | 13.5 (7.5 - 22.5) | NS |
| PC_20_ (methacholine) | 1.4 (0.7 - 2.7) | 1.3 (0.1 - 11.6) | NS |
| Serum IgE (EU/mL)^¢^ | 139.5 (42.8 - 351.0) | 198.5 (95.0 - 311.5) | NS |
| No. of positive sIgE | 6 (2 - 6) | 6 (3 - 10) | NS |
| Allergic rhinitis (%) | 33% | 72% | NS^€^ |
| Blood neutrophils (%) | 53.9 (48.0 - 61.3) | 51.7 (47.0 - 56.5) | NS |
| Blood eosinophils (%) | 3.4 (1.8 - 4.8) | 4.4 (2.5 - 9.0) | NS |
| Sputum neutrophils (%) | 49.8 (25.2 - 59.9) | 60.4 (31.0 - 66.4) | NS |
| Sputum eosinophils (%) | 0.5 (0.1 - 1.0) | 1.8 (0.3 - 5.0) | NS |
| BAL GM-CSF (pg/mL) | 292 (158 - 822) | 413 (187 - 618) | NS |
| BAL IL6 (pg/mL) | 50 (11 - 78) | 144 (62 - 372) | **0.043** |
| BAL IL7 (pg/mL) | 0.3 (0.3 - 0.3) | 14.0 (0.3 - 84.2) | **0.028** |
| BAL IL8 (pg/mL) | 1175 (377 - 1477) | 1582 (571 - 4496) | NS |
| BAL CXCL11 (pg/mL) | 140 (90 - 635) | 398 (157 - 459) | NS |
| BAL MIP-1α (pg/mL) | 39 (1 - 98) | 101 (71 - 153) | *0.067* |
| BAL MIP-1β (pg/mL) | 181 (33 - 331) | 336 (255 - 646) | *0.098* |
| BAL MIP-3α (pg/mL) | 286 (137 - 442) | 575 (255 - 1461) | NS |
| BAL TNF (pg/mL) | 40 (3 - 85) | 82 (20 - 116) | NS |
| BAL IL-1β (pg/mL) | 0.04 (0.04 - 1.0) | 0.6 (0.04 - 14.9) | NS |
| BAL IL21 (pg/mL) | 0.04 (0.04 - 0.8) | 13.9 (1.8 - 46.2) | **0.019** |

All values are medians (IQR).*ACQ - Asthma Control Questionnaire. ^¥^Alb - Albuterol ^¢^Number of positive specific IgE (sIgE >0.35 kU/l) from a total of 12 aeroallergens tested by ImmunoCap assay. Statistical significance was determined using Mann-Whitney or ^€^ Fisher’s exact test with p-values >0.1 assigned NS.

**Table S6** Specific bacterial taxa relatively enriched or depleted in nasal brush samples of asthmatic subjects whose nasal microbiota was dominated by *Corynebacterium* or other bacterial genera.

| **OTU** | **NB-C**^¥^ | **NB-nC**^€^ | **NB**  **C-nC^#^** | **Model*** | **q-value** | **Phylum** | **Order** | **Family** | **Genus** | **Species** |
| --- | --- | --- | --- | --- | --- | --- | --- | --- | --- | --- |
| 441265 | 12013.7 | 2397.0 | **9616.7** | NB | 0.025 | *Actinobacteria* | *Actinomycetales* | *Corynebacteriaceae* | *Corynebacterium* | *-* |
| 1069816 | 4733.8 | 719.9 | **4013.9** | NB | 0.080 | *Actinobacteria* | *Actinomycetales* | *Corynebacteriaceae* | *Corynebacterium* | *-* |
| 529 | 270.5 | 28.1 | **242.4** | NB | 0.017 | *Actinobacteria* | *Actinomycetales* | *Corynebacteriaceae* | *Corynebacterium* | *-* |
| 544480 | 110.0 | 16.5 | **93.5** | NB | 0.004 | *Actinobacteria* | *Actinomycetales* | *Corynebacteriaceae* | *Corynebacterium* | *-* |
| 900973 | 93.3 | 0.1 | **93.2** | NB | 0.010 | *Proteobacteria* | *Neisseriales* | *Neisseriaceae* | *-* | *-* |
| 1109 | 43.3 | 3.4 | **39.9** | NB | 1.57E^-05^ | *Actinobacteria* | *Actinomycetales* | *Corynebacteriaceae* | *Corynebacterium* | *-* |
| 128382 | 16.5 | 0.4 | **16.1** | ZINB | 0.020 | *Firmicutes* | *Clostridiales* | *Veillonellaceae* | *-* | *-* |
| 1099802 | 14.6 | 1.6 | **13.0** | NB | 0.010 | *Actinobacteria* | *Actinomycetales* | *Propionibacteriaceae* | *Propionibacterium* | *acnes* |
| 505565 | 12.3 | 0.4 | **11.9** | ZINB | 0.001 | *Firmicutes* | *Clostridiales* | *[Tissierellaceae]* | *Anaerococcus* | *-* |
| 861807 | 9.9 | 1.3 | **8.7** | NB | 0.021 | *Actinobacteria* | *Actinomycetales* | *Corynebacteriaceae* | *Corynebacterium* | *-* |
| 566578 | 5.8 | 2.9 | **2.9** | ZINB | 0.060 | *Proteobacteria* | *Burkholderiales* | *Comamonadaceae* | *Limnobacter* | *-* |
| 67 | 3.5 | 1.0 | **2.5** | ZINB | 0.084 | *Actinobacteria* | *Actinomycetales* | *Corynebacteriaceae* | *Corynebacterium* | *-* |
| 1108289 | 2.4 | 0.3 | **2.2** | ZINB | 0.010 | *Proteobacteria* | *Rhodobacterales* | *Rhodobacteraceae* | *Paracoccus* | *-* |
| 593803 | 2.2 | 0.6 | **1.6** | ZINB | 1.99E^-05^ | *Firmicutes* | *Lactobacillales* | *Streptococcaceae* | *Streptococcus* | *-* |
| 1033018 | 1.5 | 0.3 | **1.3** | ZINB | 0.006 | *Proteobacteria* | *Burkholderiales* | *Oxalobacteraceae* | *-* | *-* |
| 839235 | 1.1 | 0.6 | **0.5** | ZINB | 0.066 | *Proteobacteria* | *Aeromonadales* | *Aeromonadaceae* | *-* | *-* |
| 703741 | 1.7 | 1.4 | **0.3** | ZINB | 0.002 | *Firmicutes* | *Lactobacillales* | *Lactobacillaceae* | *Lactobacillus* | *-* |
| 1834768 | 1.4 | 2.6 | **-1.2** | ZINB | 0.020 | *Proteobacteria* | *Xanthomonadales* | *Xanthomonadaceae* | *Stenotrophomonas* | *-* |
| 153978 | 0.5 | 2.1 | **-1.6** | ZINB | 3.52E^-04^ | *Cyanobacteria* | *Streptophyta* | *-* | *-* | *-* |
| 4312974 | 0.6 | 3.3 | **-2.7** | ZINB | 0.005 | *Firmicutes* | *Bacillales* | *Staphylococcaceae* | *Staphylococcus* | *-* |
| 537430 | 1.5 | 5.8 | **-4.3** | ZINB | 1.16E^-11^ | *[Thermi]* | *Thermales* | *Thermaceae* | *Thermus* | *-* |
| 898309 | 1.0 | 5.9 | **-4.9** | ZINB | 3.52E^-04^ | *Bacteroidetes* | *Bacteroidales* | *Prevotellaceae* | *Prevotella* | *nanceiensis* |
| 341460 | 0.2 | 7.3 | **-7.1** | ZINB | 1.31E^-04^ | *Proteobacteria* | *Pasteurellales* | *Pasteurellaceae* | *Haemophilus* | *parainfluenzae* |
| 940702 | 0.1 | 7.6 | **-7.5** | NB | 0.033 | *Proteobacteria* | *Pseudomonadales* | *Moraxellaceae* | *Moraxella* | *-* |
| 1047124 | 0.6 | 10.4 | **-9.8** | ZINB | 1.56E^-04^ | *Proteobacteria* | *Pseudomonadales* | *Moraxellaceae* | *Moraxella* | *-* |
| 495396 | 0.4 | 12.5 | **-12.1** | ZINB | 3.22E^-10^ | *Firmicutes* | *Clostridiales* | *[Tissierellaceae]* | *Anaerococcus* | *-* |
| 1080004 | 1.4 | 16.1 | **-14.7** | NB | 0.020 | *Proteobacteria* | *Pseudomonadales* | *Moraxellaceae* | *Moraxella* | *-* |
| 556126 | 2.8 | 18.0 | **-15.2** | ZINB | 2.95E^-27^ | *Bacteroidetes* | *Bacteroidales* | *Bacteroidaceae* | *Bacteroides* | *-* |
| 714766 | 1.9 | 17.1 | **-15.2** | ZINB | 0.006 | *Firmicutes* | *Clostridiales* | *Lachnospiraceae* | *Moryella* | *-* |
| 716006 | 0.1 | 22.1 | **-22.0** | NB | 0.034 | *Firmicutes* | *Lactobacillales* | *Streptococcaceae* | *Lactococcus* | *-* |
| 580008 | 0.6 | 25.8 | **-25.2** | ZINB | 6.87E^-20^ | *Firmicutes* | *Erysipelotrichales* | *Erysipelotrichaceae* | *-* | *-* |
| 4310396 | 1.7 | 34.8 | **-33.1** | ZINB | 0.079 | *Bacteroidetes* | *Bacteroidales* | *[Paraprevotellaceae]* | *[Prevotella]* | *-* |
| 2195 | 2.8 | 37.3 | **-34.5** | ZINB | 0.001 | *Bacteroidetes* | *Bacteroidales* | *Prevotellaceae* | *Prevotella* | *nigrescens* |
| 851822 | 4.7 | 66.6 | **-61.9** | ZINB | 9.27E^-07^ | *Bacteroidetes* | *Bacteroidales* | *Prevotellaceae* | *Prevotella* | *-* |
| 897109 | 0.7 | 81.6 | **-80.9** | ZINB | 0.070 | *Proteobacteria* | *Pasteurellales* | *Pasteurellaceae* | *Haemophilus* | *parainfluenzae* |
| 561537 | 1.5 | 88.0 | **-86.5** | ZINB | 7.12E^-83^ | *Firmicutes* | *Clostridiales* | *Veillonellaceae* | *Selenomonas* | *-* |
| 579608 | 7.0 | 130.0 | **-123.0** | NB | 0.084 | *Firmicutes* | *Lactobacillales* | *Streptococcaceae* | *Streptococcus* | *-* |
| 1059729 | 2.8 | 129.8 | **-127.0** | NB | 0.094 | *Firmicutes* | *Lactobacillales* | *-* | *-* | *-* |
| 769222 | 0.9 | 154.4 | **-153.5** | NB | 0.005 | *Cyanobacteria* | *Streptophyta* | *-* | *-* | *-* |
| 4307391 | 8.2 | 165.0 | **-156.8** | ZINB | 0.018 | *Bacteroidetes* | *Bacteroidales* | *Prevotellaceae* | *Prevotella* | *melaninogenica* |
| 708680 | 3.5 | 190.9 | **-187.4** | ZINB | 0.020 | *Firmicutes* | *Clostridiales* | *-* | *-* | *-* |
| 1131894 | 1.3 | 194.1 | **-192.8** | ZINB | 0.040 | *Cyanobacteria* | *Streptophyta* | *-* | *-* | *-* |
| 2307137 | 5.2 | 624.1 | **-618.9** | NB | 0.032 | *Cyanobacteria* | *Streptophyta* | *-* | *-* | *-* |
| 579141 | 3.1 | 852.8 | **-849.7** | ZINB | 0.078 | *Proteobacteria* | *Pasteurellales* | *Pasteurellaceae* | *Aggregatibacter* | *-* |
| 761024 | 10.2 | 1356.8 | **-1346.6** | NB | 0.083 | *Proteobacteria* | *Neisseriales* | *Neisseriaceae* | *-* | *-* |
| 1083037 | 637.1 | 5867.1 | **-5230.0** | NB | 0.022 | *Proteobacteria* | *Pseudomonadales* | *Moraxellaceae* | *Moraxella* | *-* |

^¥^Nasal microbiota dominated by *Corynebacterium* ^€^Nasal microbiota dominated by non-*Corynebacterium* **^#^**Difference in mean relative abundance between groups; Significance was determined using *NB-Negative Binomial, PSN- Poisson, ZINB-Zero Inflated Negative Binomial regression models and corrected for false discovery, q<0.1.

**Table S7** Specific bacterial taxa relatively enriched or depleted in bronchial brush samples of asthmatic subjects whose nasal microbiota was dominated by *Corynebacterium* or other bacterial genera.

| **OTU** | **NB-C**^¥^ | **NB-nC**^€^ | **NB**  **C-nC^#^** | **Model*** | **q-value** | **Phylum** | **Order** | **Family** | **Genus** | **Species** |
| --- | --- | --- | --- | --- | --- | --- | --- | --- | --- | --- |
| **12297** | 2596.4 | 0.1 | **2596.3** | ZINB | 0.009 | *Actinobacteria* | *Actinomycetales* | *Microbacteriaceae* | *-* | *-* |
| **1107027** | 10.9 | 2.1 | **8.8** | ZINB | 1.81E^-17^ | *Firmicutes* | *Lactobacillales* | *Lactobacillaceae* | *Lactobacillus* | *-* |
| **872** | 8.1 | 3.0 | **5.1** | ZINB | 3.13E^-11^ | *Fusobacteria* | *Fusobacteriales* | *Leptotrichiaceae* | *Leptotrichia* | *-* |
| **1400** | 5.2 | 1.1 | **4.1** | ZINB | 4.82E^-08^ | *Fusobacteria* | *Fusobacteriales* | *Leptotrichiaceae* | *Leptotrichia* | *-* |
| **851923** | 4.1 | 0.2 | **3.9** | ZINB | 0.028 | *Bacteroidetes* | *Bacteroidales* | *Prevotellaceae* | *Prevotella* | *-* |
| **245523** | 5.1 | 1.8 | **3.3** | ZINB | 0.028 | *Bacteroidetes* | *Bacteroidales* | *-* | *-* | *-* |
| **1136** | 4.0 | 2.2 | **1.8** | ZINB | 0.001 | *Fusobacteria* | *Fusobacteriales* | *Leptotrichiaceae* | *Leptotrichia* | *-* |
| **563798** | 0.4 | 1.7 | **-1.2** | ZINB | 0.000 | *Fusobacteria* | *Fusobacteriales* | *Leptotrichiaceae* | *Leptotrichia* | *-* |
| **4308098** | 0.7 | 2.4 | **-1.8** | ZINB | 0.004 | *Fusobacteria* | *Fusobacteriales* | *Leptotrichiaceae* | *Leptotrichia* | *-* |
| **543032** | 0.2 | 3.1 | **-2.9** | ZINB | 0.002 | *Bacteroidetes* | *Bacteroidales* | *Prevotellaceae* | *Prevotella* | *-* |
| **236910** | 0.3 | 3.4 | **-3.1** | ZINB | 0.001 | *Proteobacteria* | *Pasteurellales* | *Pasteurellaceae* | *Actinobacillus* | *porcinus* |
| **785526** | 0.3 | 4.2 | **-3.9** | ZINB | 0.009 | *Proteobacteria* | *Rhizobiales* | *Methylobacteriaceae* | *-* | *-* |
| **679245** | 0.1 | 4.6 | **-4.4** | ZINB | 0.040 | *Firmicutes* | *Lactobacillales* | *Lactobacillaceae* | *Lactobacillus* | *zeae* |
| **827497** | 0.1 | 5.6 | **-5.4** | ZINB | 0.025 | *Proteobacteria* | *Pseudomonadales* | *Pseudomonadaceae* | *Pseudomonas* | *-* |
| **4377731** | 1.0 | 7.3 | **-6.3** | ZINB | 4.96E^-05^ | *Proteobacteria* | *Neisseriales* | *Neisseriaceae* | *Neisseria* | *-* |
| **4477971** | 0.3 | 6.8 | **-6.4** | ZINB | 0.003 | *Firmicutes* | *Clostridiales* | *-* | *-* | *-* |
| **851824** | 1.7 | 8.3 | **-6.7** | ZINB | 0.001 | *Firmicutes* | *Erysipelotrichales* | *Erysipelotrichaceae* | *Bulleidia* | *-* |
| **4419379** | 0.7 | 7.8 | **-7.1** | ZINB | 0.022 | *Bacteroidetes* | *Bacteroidales* | *[Paraprevotellaceae]* | *[Prevotella]* | *-* |
| **1077185** | 1.6 | 11.9 | **-10.3** | ZINB | 1.24E^-12^ | *Bacteroidetes* | *Bacteroidales* | *-* | *-* | *-* |
| **553611** | 1.1 | 12.9 | **-11.8** | ZINB | 3.21E^-22^ | *Actinobacteria* | *Bifidobacteriales* | *Bifidobacteriaceae* | *Bifidobacterium* | *-* |
| **545299** | 1.4 | 13.2 | **-11.8** | NB | 0.040 | *Fusobacteria* | *Fusobacteriales* | *Fusobacteriaceae* | *Fusobacterium* | *-* |
| **3859** | 0.3 | 12.8 | **-12.4** | ZINB | 0.037 | *Spirochaetes* | *Spirochaetales* | *Spirochaetaceae* | *Treponema* | *amylovorum* |
| **362965** | 0.2 | 14.7 | **-14.4** | ZINB | 4.56E^-08^ | *Actinobacteria* | *Actinomycetales* | *Micrococcaceae* | *-* | *-* |
| **250288** | 0.9 | 15.7 | **-14.8** | ZINB | 7.71E^-14^ | *Firmicutes* | *Clostridiales* | *Peptostreptococcaceae* | *-* | *-* |
| **4300564** | 0.2 | 15.1 | **-14.9** | ZINB | 4.00E^-08^ | *Proteobacteria* | *Caulobacterales* | *Caulobacteraceae* | *Asticcacaulis* | *biprosthecium* |
| **4303761** | 0.6 | 21.6 | **-21.0** | ZINB | 0.004 | *Bacteroidetes* | *Bacteroidales* | *-* | *-* | *-* |
| **249740** | 0.3 | 23.4 | **-23.1** | ZINB | 4.93E^-09^ | *Bacteroidetes* | *Flavobacteriales* | *Flavobacteriaceae* | *Capnocytophaga* | *-* |
| **225284** | 0.6 | 28.9 | **-28.3** | ZINB | 6.39E^-15^ | *Proteobacteria* | *Pseudomonadales* | *Pseudomonadaceae* | *-* | *-* |
| **862115** | 5.9 | 44.7 | **-38.8** | NB | 0.052 | *Proteobacteria* | *Pasteurellales* | *Pasteurellaceae* | *Aggregatibacter* | *-* |
| **42091** | 7.7 | 53.6 | **-45.9** | ZINB | 0.041 | *Firmicutes* | *Clostridiales* | *Peptococcaceae* | *Peptococcus* | *-* |
| **74330** | 7.2 | 59.0 | **-51.8** | NB | 0.004 | *Spirochaetes* | *Spirochaetales* | *Spirochaetaceae* | *Treponema* | *socranskii* |
| **217734** | 9.7 | 74.6 | **-64.9** | ZINB | 0.009 | *Firmicutes* | *Lactobacillales* | *Streptococcaceae* | *Streptococcus* | *anginosus* |
| **1060441** | 4.8 | 129.6 | **-124.8** | ZINB | 0.082 | *Bacteroidetes* | *Bacteroidales* | *Prevotellaceae* | *Prevotella* | *-* |

^¥^Nasal microbiota dominated by *Corynebacterium* ^€^Nasal microbiota dominated by non-*Corynebacterium* **^#^**Difference in mean relative abundance between groups; Significance was determined using *NB-Negative Binomial, PSN- Poisson, ZINB-Zero Inflated Negative Binomial regression models and corrected for false discovery, q<0.1.

**Table S8** Bacterial taxa detected in the negative control and subsequently removed from the working dataset.

| **OTU** | **Phylum** | **Class** | **Order** | **Family** | **Genus** | **Species** |
| --- | --- | --- | --- | --- | --- | --- |
| 585419 | *Firmicutes* | *Clostridia* | *Clostridiales* | *Veillonellaceae* | *Veillonella* | *dispar* |
| 559527 | *Actinobacteria* | *Actinobacteria* | *Bifidobacteriales* | *Bifidobacteriaceae* | *Bifidobacterium* | *-* |
| 192963 | *Verrucomicrobia* | *Verrucomicrobiae* | *Verrucomicrobiales* | *Verrucomicrobiaceae* | *Akkermansia* | *muciniphila* |
| 514940 | *Firmicutes* | *Clostridia* | *Clostridiales* | *Ruminococcaceae* | *-* | *-* |
| 4307652 | *Bacteroidetes* | *Bacteroidia* | *Bacteroidales* | *Prevotellaceae* | *Prevotella* | *melaninogenica* |
| 1083194 | *Firmicutes* | *Bacilli* | *Lactobacillales* | *Streptococcaceae* | *Streptococcus* | *-* |
| 1074210 | *Firmicutes* | *Bacilli* | *Gemellales* | *Gemellaceae* | *-* | *-* |
| 1084952 | *Proteobacteria* | *Betaproteobacteria* | *Neisseriales* | *Neisseriaceae* | *Neisseria* | *subflava* |
| 922761 | *Proteobacteria* | *Gammaproteobacteria* | *Enterobacteriales* | *Enterobacteriaceae* | *-* | *-* |
| 583117 | *Bacteroidetes* | *Bacteroidia* | *Bacteroidales* | *Bacteroidaceae* | *Bacteroides* | *-* |
| 1029949 | *Firmicutes* | *Clostridia* | *Clostridiales* | *Lachnospiraceae* | *Lachnospira* | *-* |
| 968954 | *Firmicutes* | *Bacilli* | *Lactobacillales* | *Streptococcaceae* | *Streptococcus* | *-* |
| 1017181 | *Actinobacteria* | *Actinobacteria* | *Actinomycetales* | *Micrococcaceae* | *Rothia* | *mucilaginosa* |
| 1083508 | *Proteobacteria* | *Gammaproteobacteria* | *Xanthomonadales* | *Xanthomonadaceae* | *-* | *-* |
| 935742 | *Bacteroidetes* | *Bacteroidia* | *Bacteroidales* | *Prevotellaceae* | *Prevotella* | *melaninogenica* |
| 1042850 | *Fusobacteria* | *Fusobacteriia* | *Fusobacteriales* | *Leptotrichiaceae* | *Leptotrichia* | *-* |
| 535375 | *Bacteroidetes* | *Bacteroidia* | *Bacteroidales* | *Bacteroidaceae* | *Bacteroides* | *ovatus* |
| 557665 | *Bacteroidetes* | *Bacteroidia* | *Bacteroidales* | *Prevotellaceae* | *Prevotella* | *-* |
| 226299 | *Proteobacteria* | *Gammaproteobacteria* | *Pseudomonadales* | *Pseudomonadaceae* | *Pseudomonas* | *-* |
| 865469 | *Proteobacteria* | *Gammaproteobacteria* | *Pasteurellales* | *Pasteurellaceae* | *Haemophilus* | *-* |
| 342427 | *Firmicutes* | *Clostridia* | *Clostridiales* | *Veillonellaceae* | *Veillonella* | *dispar* |
| 938948 | *Fusobacteria* | *Fusobacteriia* | *Fusobacteriales* | *Fusobacteriaceae* | *Fusobacterium* | *-* |
| 4306836 | *Firmicutes* | *Bacilli* | *Lactobacillales* | *Streptococcaceae* | *Streptococcus* | *-* |
| 365385 | *Actinobacteria* | *Actinobacteria* | *Bifidobacteriales* | *Bifidobacteriaceae* | *Bifidobacterium* | *-* |
